# FGF1^ΔHBS^ ameliorates chronic kidney disease via PI3K/AKT mediated suppression of oxidative stress and inflammation

**DOI:** 10.1038/s41419-019-1696-9

**Published:** 2019-06-12

**Authors:** Dezhong Wang, Mengyun Jin, Xinyu Zhao, Tianyang Zhao, Wei Lin, Zhengle He, Miaojuan Fan, Wei Jin, Jie Zhou, Lingwei Jin, Chao Zheng, Hui Jin, Yushuo Zhao, Xiaokun Li, Lei Ying, Yang Wang, Guanghui Zhu, Zhifeng Huang

**Affiliations:** 1 School of Pharmaceutical Sciences & Center for Structural Biology, Wenzhou Medical University, Wenzhou, 325035 Zhejiang China; 2 School of Life and Environmental Science, Wenzhou University, Wenzhou, 325035 Zhejiang China; 3 The Second Affiliated Hospital and Yuying Children’s Hospital of Wenzhou Medical University, Wenzhou, 325035 Zhejiang China; 4 School of Basic Medical Sciences, Wenzhou Medical University, Wenzhou, 325035 Zhejiang China; 5 The First Affiliated Hospital of Wenzhou Medical University, Wenzhou, 325035 Zhejiang China

**Keywords:** Cell biology, Molecular biology

## Abstract

Currently, there is a lack of effective therapeutic approaches to the treatment of chronic kidney disease (CKD) with irreversible deterioration of renal function. This study aimed to investigate the ability of mutant FGF1 (FGF1^ΔHBS^, which has reduced mitogenic activity) to alleviate CKD and to study its associated mechanisms. We found that FGF1^ΔHBS^ exhibited much weaker mitogenic activity than wild-type FGF1 (FGF1^WT^) in renal tissues. RNA-seq analysis revealed that FGF1^ΔHBS^ inhibited oxidative stress and inflammatory signals in mouse podocytes challenged with high glucose. These antioxidative stress and anti-inflammatory activities of FGF1^ΔHBS^ prevented CKD in two mouse models: a diabetic nephropathy model and an adriamycin-induced nephropathy model. Further mechanistic analyses suggested that the inhibitory effects of FGF1^ΔHBS^ on oxidative stress and inflammation were mediated by activation of the GSK-3β/Nrf2 pathway and inhibition of the ASK1/JNK signaling pathway, respectively. An in-depth study demonstrated that both pathways are under control of PI3K/AKT signaling activated by FGF1^ΔHBS^. This finding expands the potential uses of FGF1^ΔHBS^ for the treatment of various kinds of CKD associated with oxidative stress and inflammation.

## Introduction

Chronic kidney disease (CKD) is an irreversible disease characterized by progressive deterioration of renal function and, ultimately, uremia^1^. Currently, there are few efficient therapeutic approaches to treat this disease, thereby imposing enormous health and economic burdens on patients^2^. The etiology of CKD varies, including metabolic diseases (e.g., obesity, diabetes mellitus), hypertension, and drug intoxication^3^. Furthermore, there are complicated mechanisms involved in the initiation and progression of CKD, of which inflammation and oxidative stress are regarded as two major contributors^4^.

It has been generally accepted that there is low-grade inflammation in the initial stages of CKD associated with renal dysfunction^5,6^. Subsequently, amplified and continued inflammation accompanies the progression of CKD, during which IL-6 and TNF-α remain persistently elevated^7^. Moreover, inflammation and oxidative stress are mutually induced; each worsens in the presence of the other^8,9^. For example, the activity of NF-κB, a vital transcription factor in the inflammatory response, is directly enhanced by reactive oxygen species (ROS)^10,11^. On the other hand, NF-κB induces ROS production through the regulation of gp91phox^12^. Several lines of evidence suggest that ROS levels are upregulated and the capacity of the antioxidant system is largely damaged in the early stages of CKD, even before the initiation of inflammation^4,13–15^. As with inflammation, severe oxidative stress occurs during the progression of CKD. Therefore, drugs designed to attenuate inflammation and oxidative stress are promising strategies to slow the progression of CKD.

Although regarded as a paracrine hormone involved in embryonic development, wound healing, neurogenesis, and mitogenesis, fibroblast growth factor 1 (FGF1) has recently been found to be an efficient insulin sensitizer for the alleviation of type 2 diabetes mellitus and an important factor for maintaining the normal function of adipose tissue and metabolic homeostasis^16,17^. Subsequent studies have demonstrated that the metabolic regulation of FGF1 is associated with its anti-inflammatory effects^17–19^. Wu et al.^20^ found that wild-type FGF1 administration attenuated the production of reactive oxygen and nitrogen species in *db/db* mice. However, this report did not elucidate the precise signaling cascades regulated by FGF1 related to cellular oxidative stress; the underlying mechanisms remain elusive. Therefore, further exploration of FGF1-regulated signaling pathways involved in redox homeostasis is necessary to elucidate the molecular mechanisms mediating the protective effect of FGF1 on renal function.

Wild-type FGF1-induced hyperproliferation, which leads to an increased risk of tumorigenesis^21^, has become the primary obstacle for its wide application, particularly for chronic diseases, and including CKD. Guided by detailed insights into the structure of FGFs and FGFRs, we recently engineered an FGF1 partial agonist carrying three mutations (Lys127Asp, Lys128Gln, and Lys133Val, termed FGF1^ΔHBS^) that shows decreased ability to induce heparan sulfate (HS)-assisted FGF receptor (FGFR) dimerization and activation^22^. As expected, FGF1^ΔHBS^ exhibited dramatically reduced proliferative potential with the full metabolic activity of FGF1^WT^ in vitro and in vivo^22^. In this study, we used two CKD mouse models (the diabetic nephropathy (DN)- and adriamycin (ADR)-induced nephropathy (AN) mouse models) to investigate the effects of FGF1^ΔHBS^ on CKD. We showed that both structural and functional renal deterioration in CKD mice was markedly reversed by FGF1^ΔHBS^-mediated AKT activation associated with the inhibition of apoptosis signal-regulating kinase 1 (ASK1)-mediated c-Jun N-terminal kinase (JNK) activation and the restoration of cellular redox homeostasis via the GSK-3β/Nuclear factor erythroid-2-related factor 2 (Nrf2) signaling cascade.

## Results

### rFGF1^ΔHBS^ shows reduced proliferative activity

**Fig. 1:**
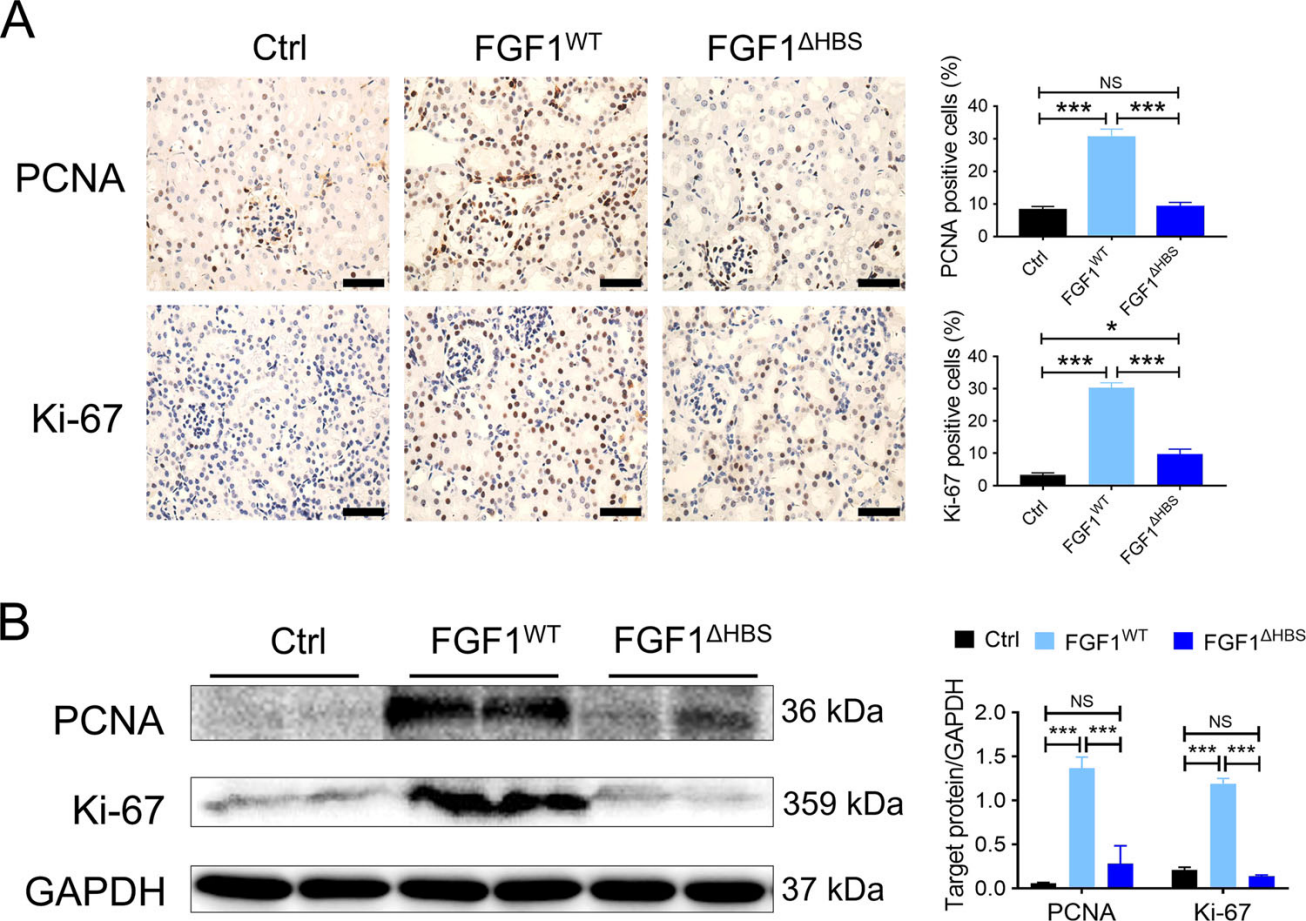
rFGF1^ΔHBS^ exhibits reduced mitogenic activity in renal tissue compared to rFGF1^WT^. C57BL/6J mice after 3 weeks of chronic administration of rFGF1 ^WT^ (2 mg/kg body weight), rFGF1^ΔHBS^ (2 mg/kg body weight), or control vehicle. **a** Representative images of PCNA or Ki-67 immunohistochemical staining of renal tissues (left panel) and quantitation using ImageJ software (right panel). Scale bar, 50 μm. **b** Expression of PCNA and Ki-67 in renal tissue as measured by western blot analyses (left panel) and quantitation using ImageJ software (right panel). Data are presented as the mean ± SEM (*n* = 6). *p < 0.05, ***p *<* 0.001

We first studied the proliferative state of kidney tissue from normal mice treated with recombinant wild-type human FGF1 (rFGF1^WT^) or rFGF1^ΔHBS^ at a dose of 2 mg/kg body weight for 3 weeks. Kidney tissues were isolated and stained for PCNA or Ki-67. Immunohistochemical analyses showed that rFGF1^WT^ induced a large increase in PCNA- and Ki-67-positive cells that were largely abolished in rFGF1^ΔHBS^-treated mice (Fig. 1a). Consistent with this result, protein expression levels of PCNA and Ki-67 were much higher in renal tissues of rFGF1^WT^-treated mice, with only minimal upregulation in rFGF1^ΔHBS^-treated mice **(**Fig. 1b). These data suggest that structure-based FGF1 mutants with reduced mitogenic activity may be appropriate for the treatment of CKD.

### rFGF1^ΔHBS^ ameliorates renal dysfunction in T2D mice

**Fig. 2:**
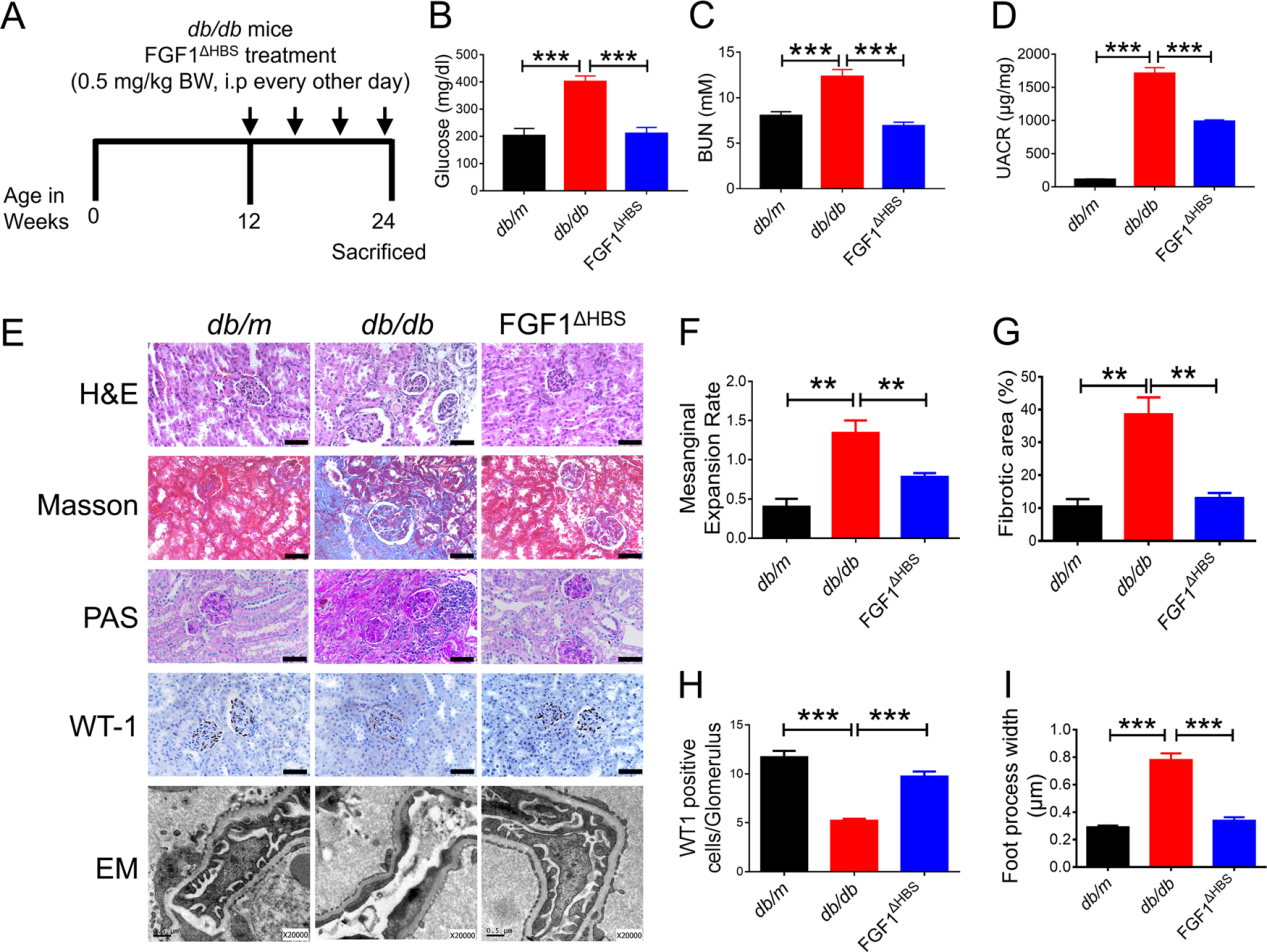
rFGF1^ΔHBS^ ameliorates diabetic nephropathy in *db/db* mice. **a** Schematic diagram of the chronic rFGF1^ΔHBS^ treatment schedule for *db/db* mice. **b**–**i** *db/db* mice were treated with rFGF1^ΔHBS^ (0.5 mg/kg body weight) or buffer control for 12 weeks; littermate *db/m* mice served as additional controls. **b**–**d** Blood glucose levels (**b**), blood urea nitrogen (BUN) levels (**c**) and urine albumin-to-creatinine ratio (UACR) (**d**) in each group. **e** Representative images of hematoxylin and eosin (H&E) staining, Masson’s trichrome staining, periodic acid Schiff (PAS) staining, immunohistochemistry staining with WT-1 antiserum, and electron microscopy (EM) images of renal tissues in each group. Scale bar, 50 μm for H&E, Masson’s trichrome, PAS, and WT-1 staining images; Scale bar, 0.5 μm for EM images. **f–i** Quantification of mesangial expansion (**f**), fibrosis area (**g**), WT-1-positive cells (**h**), and podocyte foot process effacement (**i**) in renal tissues from each group. Data are presented as the mean ± SEM (*n* = 5). **p *<* 0.01, ***p *<* 0.001

DN accounts for 50% of end-stage renal disease cases^2,23^. We assessed the protective effects of rFGF1^ΔHBS^ on T2D-induced DN according to the protocol detailed in the methods section (Fig. 2a), in which rFGF1^ΔHBS^ (0.5 mg/kg body weight) was injected intraperitoneally (i.p.) into *db/db* mice every other day for 12 weeks. Consistent with the findings in our previous study^22^, blood glucose levels (a major risk factor for DN) in *db/db* mice were markedly reduced by rFGF1^ΔHBS^ (Fig. 2b). Serum levels of blood urea nitrogen (BUN) (a marker of renal injury) were largely reduced (Fig. 2c), and the aberrant glomerular filtration rate (GFR) (estimated by the urine albumin-to-creatinine ratio) was restored in rFGF1^ΔHBS^-treated *db/db* mice (Fig. 2d). Then, histological analyses were performed to assess the protective role of rFGF1^ΔHBS^ in the structural remodeling of the kidney. Hematoxylin and eosin (H&E), Masson’s trichrome, and periodic acid Schiff (PAS) staining showed that mesangial expansion, renal fibrosis, and glycogen content were markedly reduced by rFGF1^ΔHBS^ treatment (Fig. 2e–g). Immunohistochemistry showed that the loss of Wilms’ tumor 1 (WT-1)-positive cells (a podocyte biomarker^24–26^) in *db/db* mice was rescued by rFGF1^ΔHBS^, indicating that the primary renal podocyte lesions and related dysfunction were largely alleviated (Fig. 2e, h). Furthermore, electron microscopy analysis demonstrated that diabetes-induced glomerular damage (including the disruption of podocyte foot processes and basement membrane thickening) was substantially alleviated by rFGF1^ΔHBS^ (Fig. 2i). Based on these findings, FGF1^ΔHBS^ is a potential therapeutic protein with considerable ability to improve DN, primarily by protecting against podocyte injury. Furthermore, as observed in normal mice, *db/db* mice treated for 12 consecutive weeks with rFGF1^ΔHBS^ showed less mitogenic activity in renal tissues than those in the vehicle control group (Fig. S1).

### rFGF1^ΔHBS^ prevents DN-induced inflammation and oxidative stress

**Fig. 3:**
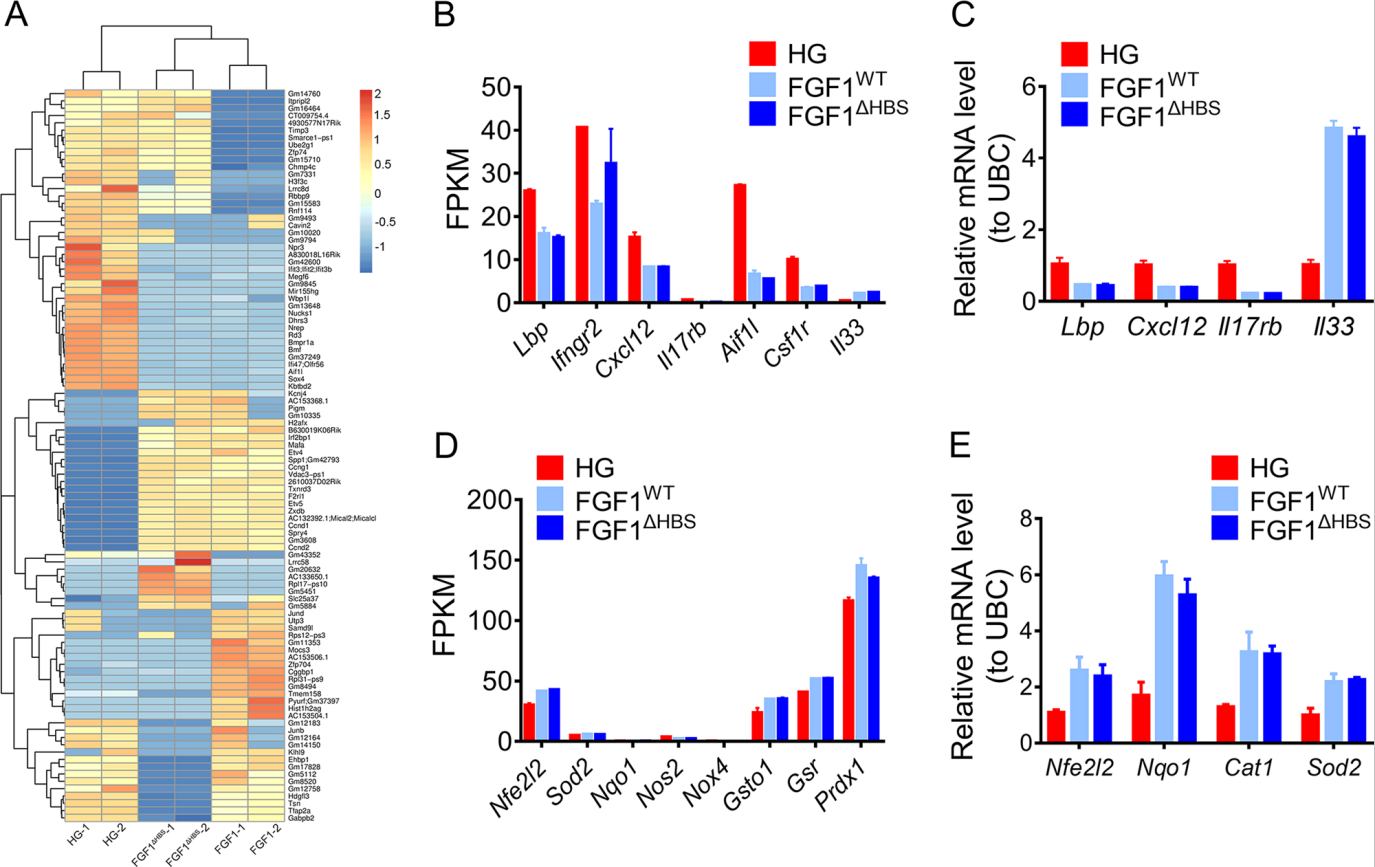
Transcriptome analysis of mouse podocytes treated with rFGF1^ΔHBS^ or rFGF1^WT^ and challenged with HG. Mouse podocytes were pretreated with rFGF1^WT^ (100 ng/mL) or rFGF1^ΔHBS^ (100 ng/mL) for 1 h, followed by treatment with HG (25 mM) for an additional 12 h. **a** Hierarchical clustering of differentially expressed genes. Red represents increased expression, while blue indicates decreased expression. The Z score was chosen to display the fragments per kilobase of exon per million reads mapped (FPKM) values of differentially expressed genes. **b** FPKM values of selected inflammatory genes. **c** Real-time PCR analysis of *Lbp*, *Cxcl12*, *Il17rb*, and *Il33* mRNA expression. **d** FPKM values of selected genes related to oxidative stress. **e** Real-time PCR analysis of *Nfe2l2*, *Nqo1*, *Cat1*, and *Sod2* mRNA expression

To elucidate the underlying mechanisms mediating the protective effect of rFGF1^ΔHBS^, RNA-sequencing (RNA-Seq) analysis was performed on mouse podocytes (MPCs) treated with rFGF1^ΔHBS^ or rFGF1^WT^ (100 ng/mL) under high-glucose (HG, 25 mM) conditions (Fig. 3a). We found that many inflammatory genes (including *Lbp*, *Ifngr2*, *Cxcl12*, and *Il17rb*) induced by HG were substantially abrogated (Fig. 3b), while antioxidative stress signals (including *Nfe2l2*, *Sod2*, *Nqo1*, and *Nox4*) induced by HG were upregulated by both rFGF1^WT^ and rFGF1^ΔHBS^ (Fig. 3d). These results were further validated using real-time PCR analysis, which showed that inflammatory and antioxidative signals were inhibited and enhanced, respectively, by rFGF1^ΔHBS^ and rFGF1^WT^ (Fig. 3c and e). Consistent with the in vivo experiments, proliferative signals were significantly lower in MPCs treated with rFGF1^ΔHBS^ than in those treated with rFGF1^WT^ (Fig. S2).

**Fig. 4:**
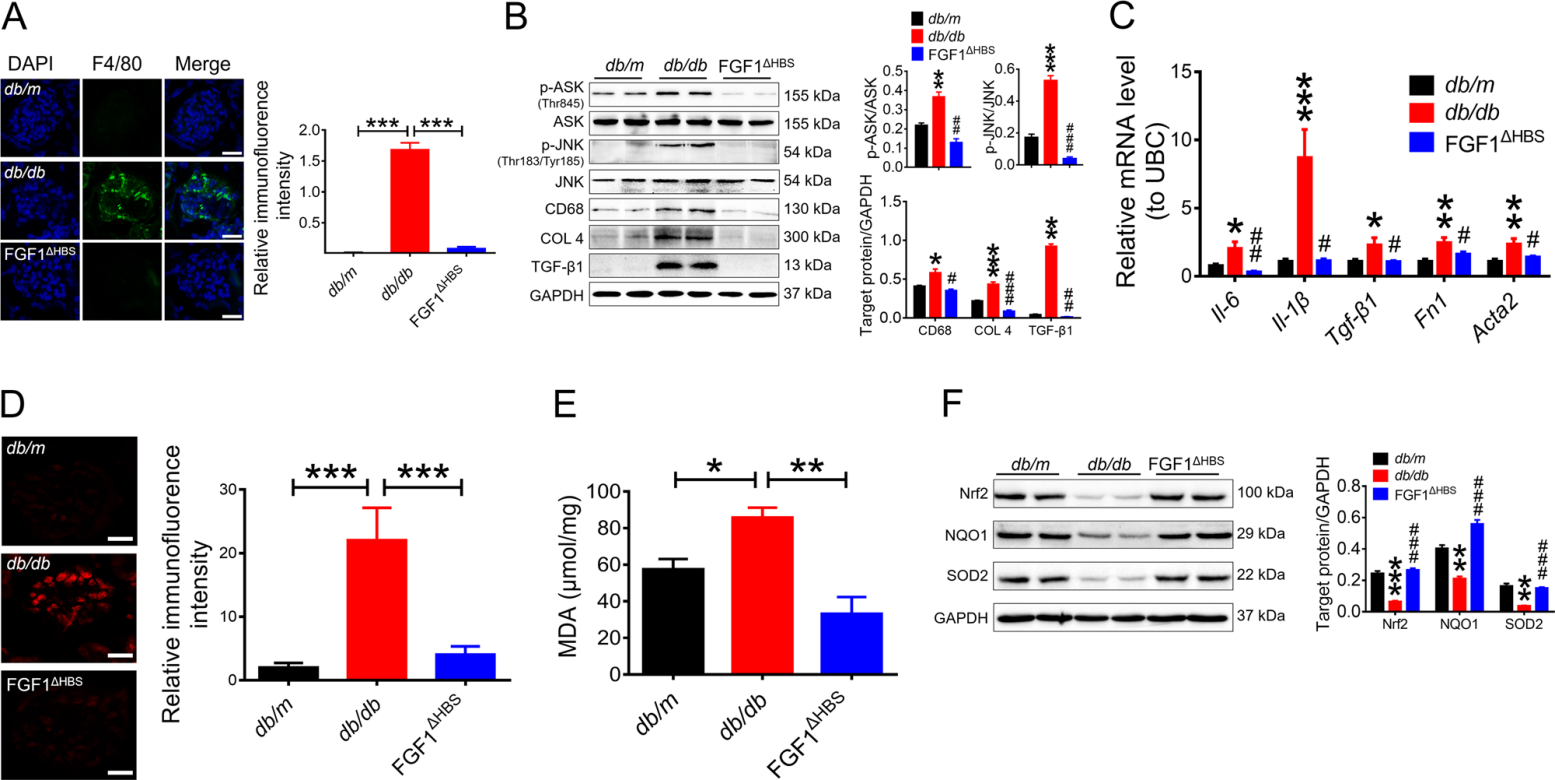
rFGF1^ΔHBS^ suppresses diabetes-induced renal inflammation and oxidative stress. Renal tissues were isolated from *db/db* mice treated for 12 weeks with rFGF1^ΔHBS^ (0.5 mg/kg body weight) or buffer control; littermate *db/m* mice served as additional controls. **a** Representative images and quantitation of immunofluorescence staining for F4/80. Scale bar, 50 μm. **b** Phosphorylation levels of ASK and JNK and expression levels of CD68, COL 4, and TGF-β1 as determined by western blot analysis (left panel) and quantitation using ImageJ software (right panel). **c** Real-time PCR analysis of *Tnf-α, IL-1β, TGF-β1, Fn1*, and *Acta2* mRNA expression. **d** Representative images and quantitation of DHE immunofluorescence. Scale bar, 50 μm. **e** ELISA analysis of MDA levels in renal tissues from each group. **f** Expression levels of Nrf2, NQO1, and SOD2 as determined by western blot analysis (left panel) and quantitation using ImageJ software (right panel). Data are presented as the mean ± SEM (*n* = 5–8). Panels **a**, **d**, **e**: *p *<* 0.05, **p *<* 0.01, ***p *<* 0.001; panels **b**, **c**, **f**: *p < 0.05, **p *<* 0.01, ***p *<* 0.001 versus *db/m*; ^#^*p* *<* 0.05, ^##^*p* *<* 0.01, ^###^*p* *<* 0.001 versus *db/db*

Given that inflammation and oxidative stress are two major risk factors for DN and are inhibited by rFGF1^ΔHBS^ in vitro, we further investigated whether these inhibitory events were involved in the protective effects of rFGF1^ΔHBS^ against DN. Large numbers of tissue macrophages have been reported to accumulate in renal tissues of T2D subjects, and substantial evidence suggests an etiological role for macrophage infiltration in the development of chronic tissue inflammation associated with DN^27–29^. Therefore, we analyzed the inflammatory status of renal tissue in rFGF1^ΔHBS^-treated *db/db* mice by immunostaining for F4/80. Immunofluorescence analyses showed substantially fewer F4/80-positive macrophages in rFGF1^ΔHBS^-treated *db/db* mice than in buffer-treated mice (Fig. 4a), consistent with the reduced protein expression of CD68 (Fig. 4b).

Macrophage-associated inflammatory responses primarily depend on activation of the NF-κB signaling pathway^30^. We found that the increased phosphorylation of ASK1 (a pivotal protein required for the activation of JNK^31,32^) and JNK in *db/db* mice was strongly suppressed by rFGF1^ΔHBS^ (Fig. 4b). Moreover, these results were associated with decreased mRNA levels of proinflammatory genes in renal tissue, including *Tnf-α and Il-1β* (Fig. 4c). In addition to reduced inflammatory responses, inflammation-associated profibrotic signals^33^ were markedly inhibited by rFGF1^ΔHBS^; specifically, the protein levels of type IV collagen (COL 4) and transforming growth factor-β 1 (TGF-β1) **(**Fig. 4b**)** and the mRNA levels of *Tgf-β1*, *Fn1*, and *Acta2* (Fig. 4c) were substantially reduced.

Oxidative stress and inflammation are mutually induced to exacerbate severe damage in DN. Therefore, we next analyzed the oxidative status of renal tissue in rFGF1^ΔHBS^-treated *db/db* mice by immunostaining with dihydroethidium (DHE). Immunofluorescence analysis showed substantially fewer DHE-positive cells in rFGF1^ΔHBS^-treated *db/db* mice than in vehicle-treated mice (Fig. 4d); this finding paralleled the reduced MDA content in renal tissue (Fig. 4e). Nrf2 is a critical transcription factor involved in maintaining redox homeostasis^34–36^. RNA-Seq analysis showed that the mRNA levels of two major downstream targets of Nrf2, NAD(P)H dehydrogenase quinone 1 (NQO1), and superoxide dismutase-2 (SOD2), were upregulated by rFGF1^ΔHBS^ (Fig. 3d). We also found that the protein expression levels of NQO1 and SOD2 in renal tissue were markedly elevated in rFGF1^ΔHBS^-treated *db/d*b mice, accompanied by Nrf2 activation (Fig. 4f).

Taken together, these data suggest that rFGF1^ΔHBS^ may protect against DN by inhibiting inflammation through the downregulated ASK1/JNK signaling pathway and oxidative stress through the upregulated Nrf2/NQO1 and SOD2 signaling pathways.

### rFGF1^ΔHBS^ ameliorates ADR-induced nephropathy by antioxidative stress and anti-inflammatory mechanisms

**Fig. 5:**
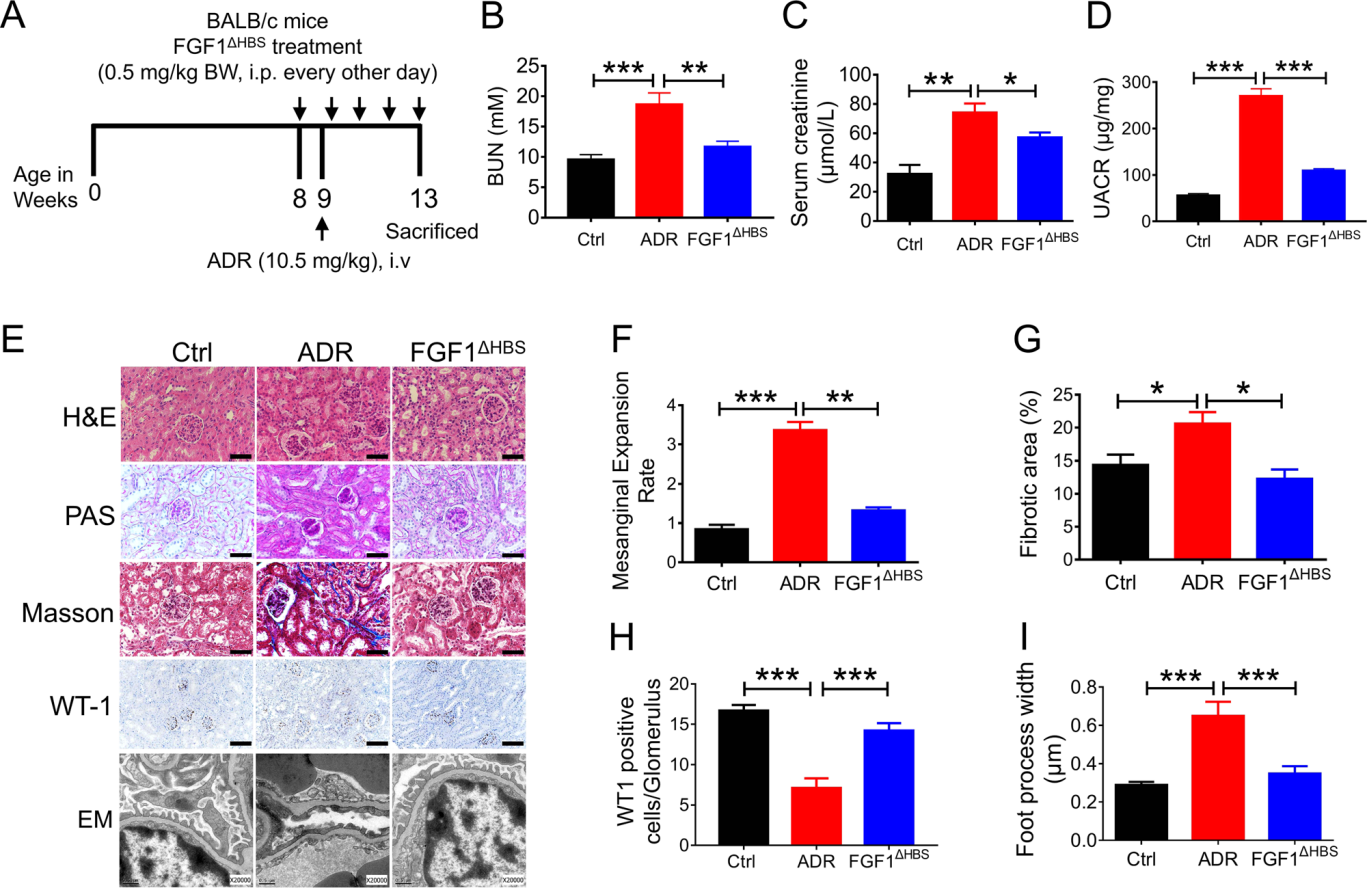
rFGF1^ΔHBS^ rescues ADR-induced nephropathy. **a** Schematic diagram of the ADR-induced (10.5 mg/kg body weight) nephropathy (AN) model and chronic rFGF1^ΔHBS^ treatment. **b–i** AN mice were treated with rFGF1^ΔHBS^ (0.5 mg/kg body weight) or buffer control for 5 weeks; normal BALB/c mice served as additional controls (Ctrl). **b–d** ELISA analysis of blood urea nitrogen (BUN) levels (**b**), creatinine levels (**c**), and UACR (**d**) in each group. **e** H&E staining, Masson’s trichrome staining, PAS staining, immunohistochemistry staining with WT-1 antiserum, and EM images of renal tissues in each group. Scale bar, 50 μm for H&E, Masson’s trichrome, PAS, and WT-1 staining images; Scale bar, 0.5 μm for EM images. **f–i** Quantification of mesangial expansion (**f**), fibrosis area (**g**), WT-1-positive cells per glomerulus (**h**), and podocyte foot process effacement (**i**) in renal tissues. Data are presented as the mean ± SEM (*n* = 5); *p *<* 0.05, **p *<* 0.01, ***p *<* 0.001

To explore whether the anti-inflammatory and antioxidative stress properties of rFGF1^ΔHBS^ are applicable to other related CKDs, an AN model that mirrors human focal segmental glomerulosclerosis (including tubulointerstitial inflammation, oxidative stress, and fibrosis)^37,38^ was generated (Fig. 5a). As previously reported^19^, FGF1 was highly expressed in the kidneys of healthy mice. Both immunofluorescence and western blotting analyses showed that the protein expression of FGF1 was substantially decreased in mice with ADR-induced nephropathy (Fig. S3A, B), suggesting a potential correlation between the levels of FGF1 and renal function. Consistent with the ability of rFGF1^ΔHBS^ to alleviate nephropathy in T2D mice, we found that serum levels of BUN and creatinine were reduced and that the abnormal GFR was restored in rFGF1^ΔHBS^-treated AN mice (Fig. 5b–d). Histological analyses also revealed that mesangial expansion (H&E staining), renal fibrosis (Masson’s trichrome staining), glycogen content (PAS staining), primary renal podocyte lesions (WT-1 staining), and foot process loss were alleviated by rFGF1^ΔHBS^ treatment (Fig. 5e–i).

**Fig. 6:**
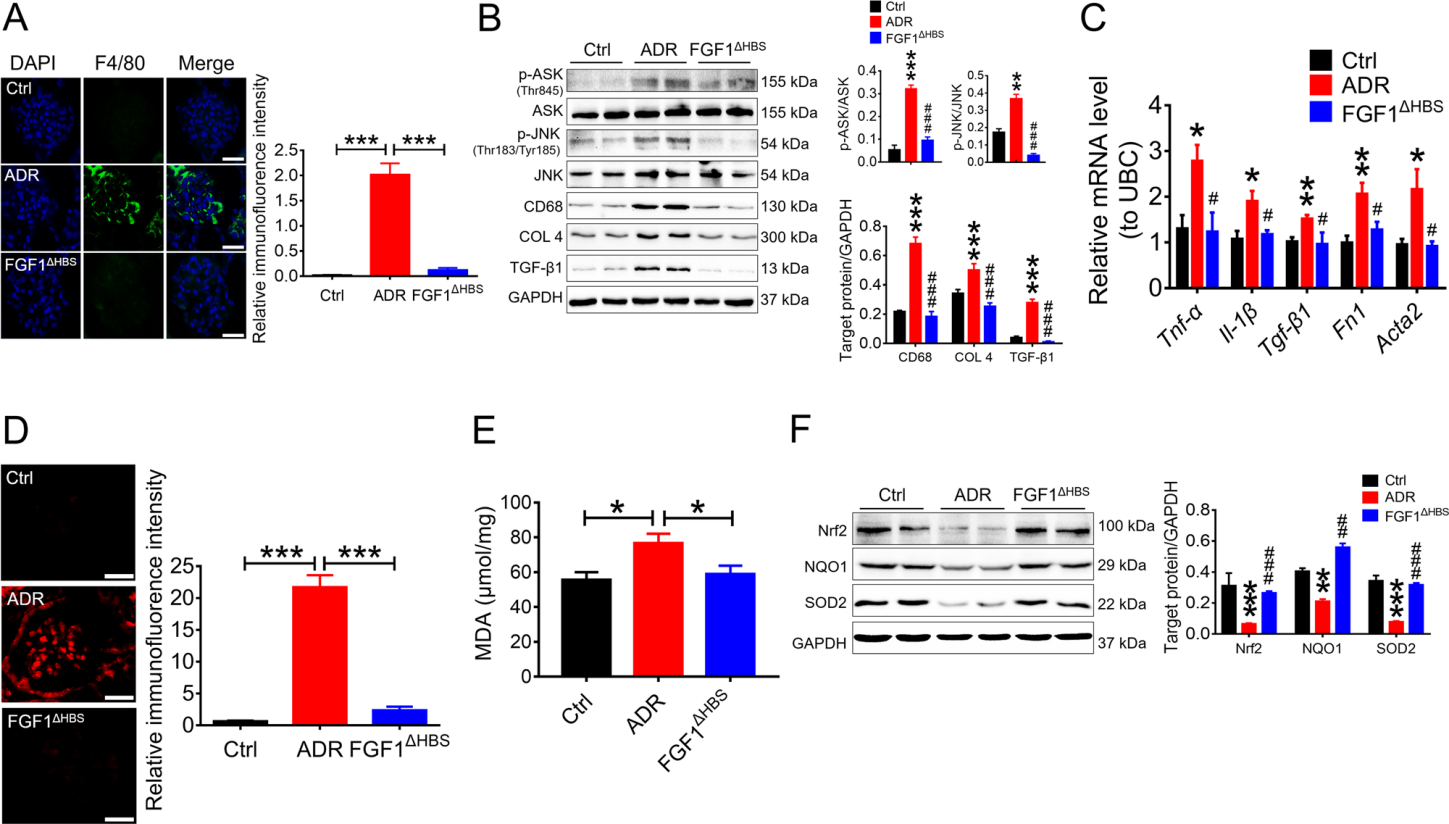
rFGF1^ΔHBS^ inhibits ADR-induced inflammation and oxidative stress in the kidney. Analysis of renal tissues from mice with ADR-induced (10.5 mg/kg body weight) nephropathy treated for 5 weeks with rFGF1^ΔHBS^ (0.5 mg/kg body weight) or buffer control; normal BALB/c mice served as additional controls. **a** Representative images and quantitation of immunofluorescence staining for F4/80. Scale bar, 50 μm. **b** Phosphorylation levels of ASK and JNK and expression levels of CD68, COL 4, and TGF-β1 as determined by western blot analysis (left panel) and quantitation using ImageJ software (right panel). **c** Real-time PCR analysis of *Tnf-α, Il-1β, Tgf-β1, Fn1, and Acta2* mRNA expression. **d** Representative images and quantitation of DHE immunofluorescence. Scale bar, 50 μm. **e** ELISA analysis of MDA levels in each group. **f** Expression of Nrf2, NQO1, and SOD2 as determined by western blot analysis (left panel) and quantitation using ImageJ software (right panel). Data are presented as the mean ± SEM (*n* = 5–8). Panels **a**, **d**, **e**: *p *<* 0.05, ***p *<* 0.001; panels **b**, **c**, **f**: *p *<* 0.05, **p < 0.01, ***p *<* 0.001 versus Ctrl; ^#^*p* *<* 0.05, ^##^*p* < 0.01, ^###^*p* *<* 0.001 versus ADR

Consistent with the finding that rFGF1^ΔHBS^ inhibited oxidative stress and inflammation in DN, we found significantly fewer F4/80-positive macrophages and lower protein expression of CD68 in rFGF1^ΔHBS^-treated AN mice than in buffer-treated mice (Fig. 6a and b). This effect was associated with reduced phosphorylation of ASK1 and JNK and decreased mRNA levels of proinflammatory genes (Fig. 6b and c). Inflammation-associated profibrotic signals were inhibited by treatment with rFGF1^ΔHBS^ (Fig. 6b and c). Furthermore, we found a significant reduction in the number of DHE-positive cells and reduced MDA content in renal tissues of rFGF1^ΔHBS^-treated AN mice, associated with elevated expression levels of Nrf2, NQO1, and SOD2 (Fig. 6d-f). Taken together, these data suggest that rFGF1^ΔHBS^ prevents inflammation- and oxidation-associated CKDs.

### The protective effects of rFGF1^ΔHBS^ on CKD is mediated by AKT activation

**Fig. 7:**
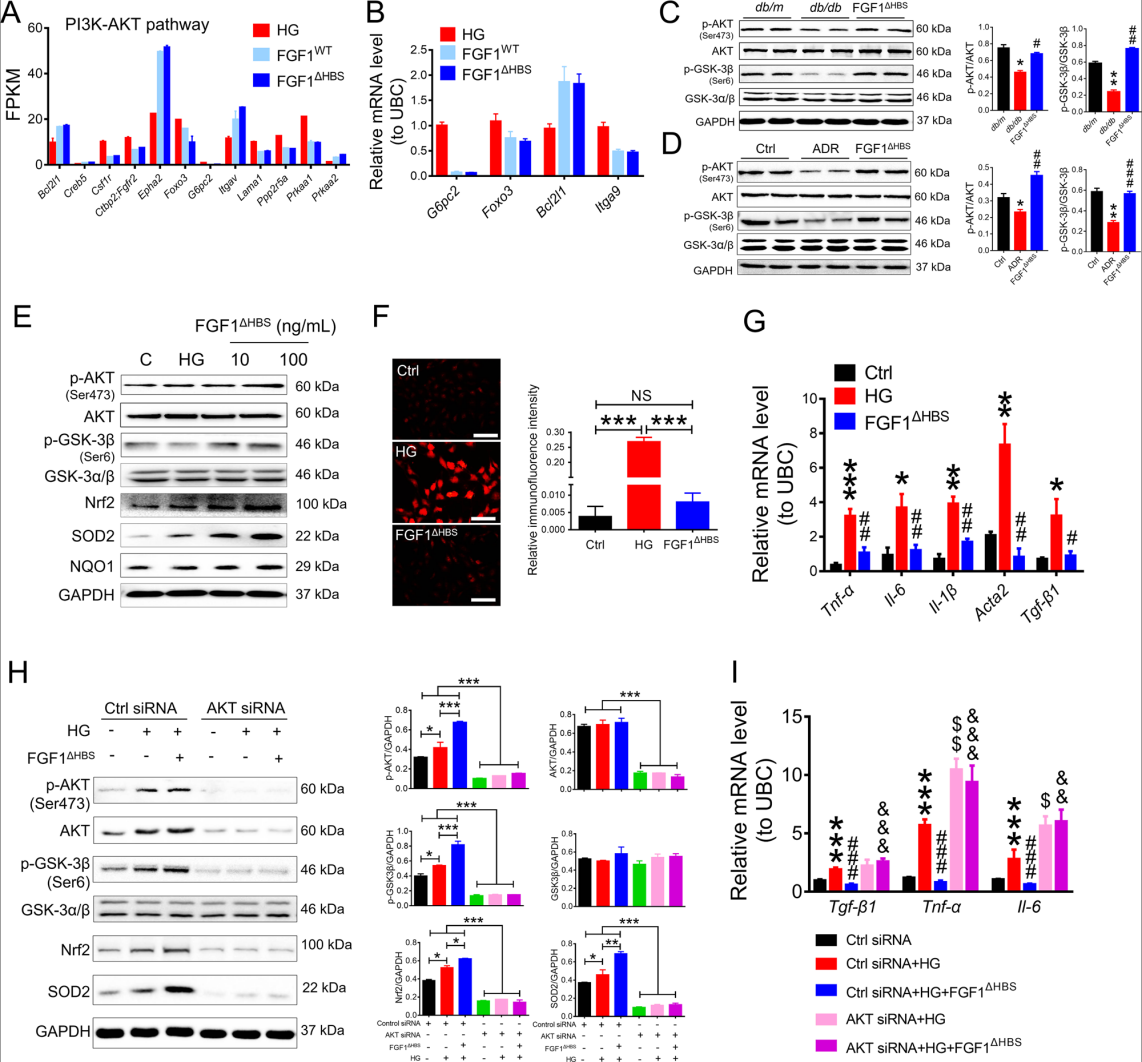
rFGF1^ΔHBS^ inhibits HG-induced inflammation and oxidative stress in mouse podocytes via AKT activation. **a** Transcriptome analysis revealed that the PI3K/AKT pathway was activated by treatment with either rFGF1^WT^ or rFGF1^ΔHBS^. **b** Real-time PCR analysis of *G6pc2*, *Foxo3*, *Bcl2l1*, and *Itga9* mRNA expression. The samples in panels **a**, **b** were the same as those in Fig. 4. Mouse podocytes were pretreated with rFGF1^WT^ (100 ng/mL) or rFGF1^ΔHBS^ (100 ng/mL) for 1 h, followed by treatment with HG (25 mM) for an additional 12 h. **c**, **d** Phosphorylation levels of AKT and GSK-3β in renal tissues from *db/db* mice after 12 consecutive weeks of injection with rFGF1^ΔHBS^ (0.5 mg/kg body weight) (**c**) or from mice with ADR-induced (10.5 mg/kg body weight) nephropathy after 5 consecutive weeks of injection with rFGF1^ΔHBS^ (0.5 mg/kg body weight) (**d**) as determined by western blotting (left panel) and quantitated using ImageJ software (right panel). Data are presented as the mean ± SEM (*n* = 8). **e**–**g** Analysis of mouse podocytes pretreated with rFGF1^ΔHBS^ (10 or 100 ng/mL for 1 h) and exposed to either low glucose (LG) or high glucose (HG, 25 mM) for an additional 12 h. **e** Phosphorylation levels of AKT and GSK-3β and protein expression levels of Nrf2, NQO1, and SOD2 as determined by western blot analysis. **f** Representative images and quantitation of DHE immunofluorescence; Scale bar, 50 μm. **g** Real-time PCR analysis of *Tnf-α, IL-1β, Tgf-β1, Fn1*, and *Acta2* mRNA expression. Data from three independent measurements are presented as the mean ± SEM. **h**, **i** Cells were transfected with control or AKT siRNA, pretreated with FGF1^ΔHBS^ (100 ng/mL) for 1 h and incubated with high glucose (25 mM) for an additional 12 h. **h** Phosphorylation levels of AKT and GSK-3β and protein expression levels of GSK-3β, Nrf2, NQO1, and SOD2 as determined by western blot analysis (left panel) and quantitation using ImageJ software (right panel). **i** Real-time PCR analysis of *TGF-β1, Tnf-α*, and *IL-6* mRNA levels. Data from three independent measurements are presented as the mean ± SEM. Panels **c**, **d**: *p *<* 0.05, **p < 0.01 versus *db/m* or Ctrl; ^#^*p* *<* 0.05, ^##^*p* *<* 0.01, ^###^*p* *<* 0.001 versus *db/db* or ADR; panel **f**: ***p < 0.001; panel **g**: *p *<* 0.05, **p *<* 0.01, ***p *<* 0.001 versus Ctrl; ^#^*p* *<* 0.05, ^##^*p* *<* 0.01 versus HG; panel **h**: *p *<* 0.05, **p *<* 0.01, ***p *<* 0.001; panel **i**: ***p *<* 0.001 versus ctrl siRNA; ^###^*p* *<* 0.001 versus ctrl siRNA + HG; ^$^*p* *<* 0.05, ^$$^*p* *<* 0.01 versus ctrl siRNA + HG; ^&&^*p* *<* 0.01, ^&&&^*p* *<* 0.001 versus ctrl siRNA + HG + FGF1^ΔHBS^

To further elucidate the regulatory mechanism of FGF1^ΔHBS^ pertaining to oxidative stress and inflammation, we revisited the RNA-Seq results. Kyoto Encyclopedia of Genes and Genomes (KEGG) analysis revealed that the phosphatidylinositide 3-kinase (PI3K)/protein kinase B (AKT) pathway was significantly upregulated by treatment with either rFGF1^ΔHBS^ or rFGF1^WT^ (Fig. 7a). This finding was further confirmed by real-time PCR analysis, which showed that rFGF1^ΔHBS^ or rFGF1^WT^ substantially reduced the mRNA levels of *G6pc2*, *Foxo3*, and *Lama1* and enhanced the expression of *Bcl2l1*, all of which are downstream targets of PI3K/AKT^39^ (Fig. 7b).

These lines of evidence suggest that AKT might be an upstream regulator of oxidative stress and inflammation in CKD. Indeed, we found that the phosphorylation levels of AKT and GSK-3β were strongly upregulated by rFGF1^ΔHBS^ in both the DN and AN mouse models (Fig. 7c, d). We also found that rFGF1^ΔHBS^ dose dependently upregulated the phosphorylation levels of AKT and GSK-3β and the protein expression levels of Nrf2, SOD2, and NQO1 in MPCs challenged with HG (Fig. 7e). This finding was consistent with the reduced number of DHE-positive cells after rFGF1^ΔHBS^ treatment (Fig. 7f). Meanwhile, the phosphorylation levels of ASK1 and JNK and the mRNA levels of proinflammatory and profibrotic genes were markedly inhibited by rFGF1^ΔHBS^ (Fig. S4A and Fig. 7g). The importance of AKT in the regulation of HG-induced inflammatory and oxidative responses in MPCs was confirmed by using siRNA and a specific inhibitor (MK-2206^40^). We found that activation of the antioxidative signaling pathway and inhibition of inflammatory and profibrotic signaling by rFGF1^ΔHBS^ were greatly inhibited by siRNA-mediated knockdown of AKT (Fig. 7h, i), and this finding was further confirmed by the dose-dependent inhibitory effect of MK-2206 **(**Fig. S4A and S5).

**Fig. 8:**
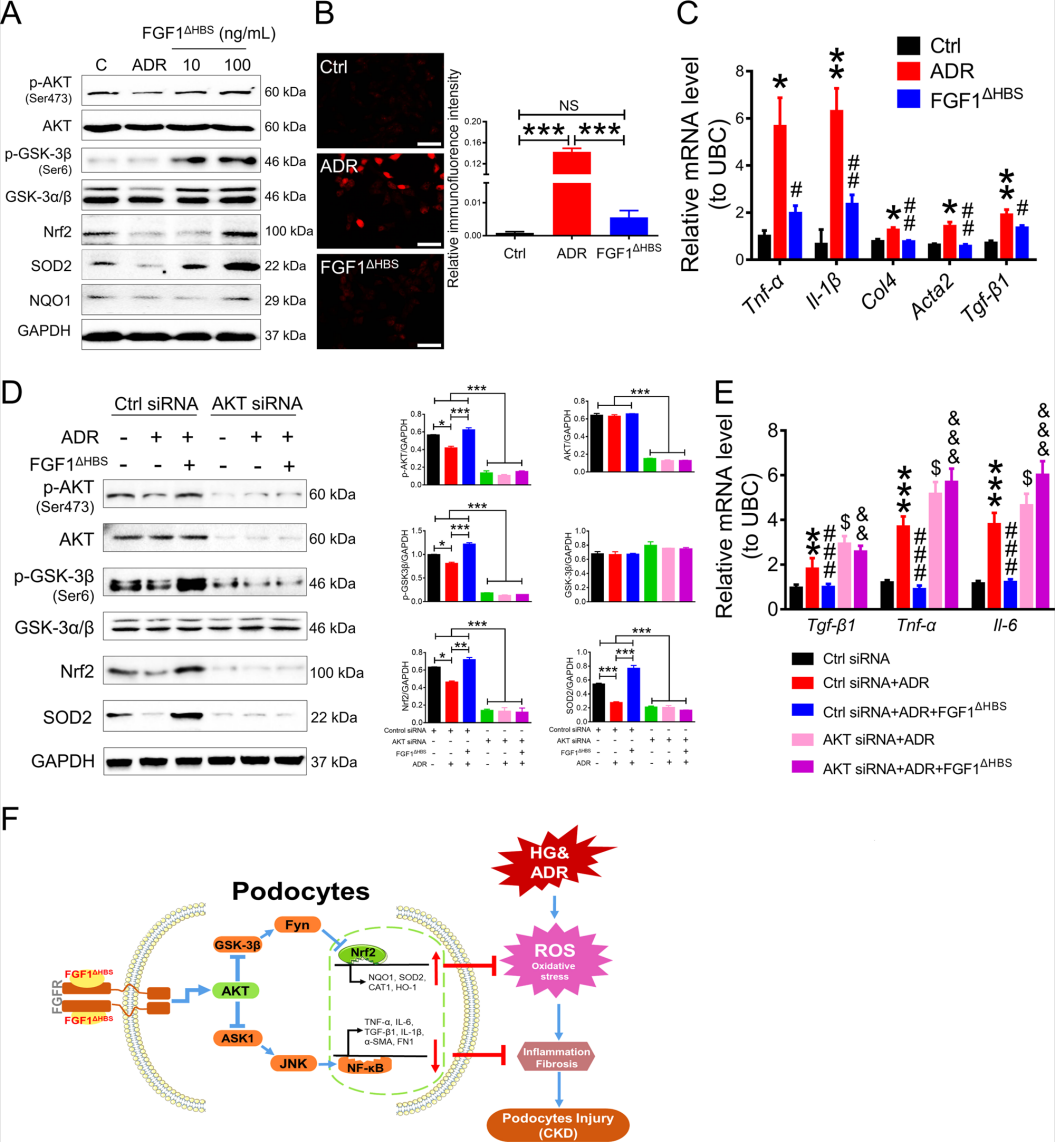
AKT mediates the protective effects of FGF1^ΔHBS^ on podocytes challenged with ADR. **a–c** Analysis of mouse podocytes pretreated with rFGF1^ΔHBS^ (10 or 100 ng/mL for 1 h) and exposed to ADR (0.5 μg/mL for 12 h). **a** Phosphorylation levels of AKT and GSK-3β and protein expression levels of Nrf2, NQO1, and SOD2 as determined by western blot analysis. **b** Representative images and quantitation of DHE immunofluorescence. Scale bar, 50 μm. **c** Real-time PCR analysis of *Tnf-α, IL-1β, TGF-β1, Fn1*, and *Acta2* mRNA expression. **d**, **e**. Cells were transfected with control or AKT siRNA, pretreated with FGF1^ΔHBS^ (100 ng/mL) for 1 h and incubated with adriamycin (0.5 μg/mL) for an additional 12 h. **d** Phosphorylation levels of AKT and GSK-3β and protein expression levels of GSK-3β, Nrf2, NQO1, and SOD2 as determined by western blot analysis (left panel) and quantitation using ImageJ software (right panel). **e** Real-time PCR analysis of *TGF-β1, Tnf-α*, and *IL-6* mRNA levels. **f** Schematic diagram of the FGF1^ΔHBS^-mediated inhibition of inflammation and oxidative stress in mouse podocytes challenged with HG or ADR. Data from three independent measurements are presented as the mean ± SEM. Panel **b**: ***p < 0.001; panel **c**: *p < 0.05, **p < 0.01 versus Ctrl; ^#^*p* < 0.05, ^##^*p* < 0.01 versus ADR; panel D: *p *<* 0.05, **p *<* 0.01, ***p *<* 0.001; panel **e**: **p *<* 0.01, ***p *<* 0.001 versus ctrl siRNA; ^###^*p* *<* 0.001 versus ctrl siRNA + ADR; ^$^*p* < 0.05 versus ctrl siRNA + ADR; ^&&^*p* *<* 0.01; ^&&&^*p* *<* 0.001 versus ctrl siRNA + ADR + FGF1^ΔHBS^

Coincidentally, ADR-induced severe oxidative stress and inflammation in MPCs were largely reversed by rFGF1^ΔHBS^; rFGF1^ΔHBS^ dose dependently upregulated the phosphorylation levels of AKT and GSK-3β and the protein expression levels of Nrf2, SOD2, and NQO1 and reduced the number of DHE-positive cells (Fig. 8a, b). Inflammatory signals (including ASK1 and JNK phosphorylation levels and the mRNA levels of proinflammatory genes) and profibrotic molecules were blunted by rFGF1^ΔHBS^ treatment (Fig. 8c and Fig. S4B). Importantly, activation of the antioxidative signaling pathway and inhibition of inflammatory and profibrotic signaling by rFGF1^ΔHBS^ were also inhibited by AKT siRNA and MK-2206 (Fig. 8d, e, Fig. S4B, S6).

Furthermore, we found that rFGF1^ΔHBS^ induced the increased phosphorylation of AKT and GSK-3β; Nrf2 and SOD2 protein levels in mesangial cells were abolished by AKT knockdown (Fig. S7). These data suggest that rFGF1^ΔHBS^ protects different types of cells from oxidative and inflammatory injury, further highlighting the importance of AKT in the protection of renal injury by inhibiting oxidative stress and inflammation.

## Discussion

Under the guidance of our FGFR dimerization threshold model, we engineered a structure-based FGF1 mutant (termed FGF1^ΔHBS^) with reduced ability to bind/dimerize cognate FGFRs. These molecules therefore had a reduced binding affinity to HS. FGF1^ΔHBS^ has been previously shown to induce transient/weak levels of receptor dimerization and correspondingly weak intracellular signals that are sufficient to mediate glucose-lowering effects but have much lower mitogenic activity^22^. Our finding that a low threshold of receptor dimerization, rather than ligand identity, is the key determinant in glucose-lowering activity implies that this action mode may be suitable to dissect other biological functions of FGF1 (that require weak activation) from its mitogenic activity (which requires strong activation). This proposal was tested in the current study, in which the capacity of FGF1 to abrogate inflammation and oxidative stress was retained in rFGF1^ΔHBS^, whereas its mitogenic activity was largely reduced.

The progression of CKD is closely related to increased inflammation and oxidative stress in renal tissue. On one hand, excessive ROS accumulation imposes a severe cytotoxic burden on the kidney, exacerbating its dysfunction^41^. On the other hand, the overproduction of inflammatory cytokines and chemokines leads to fibrosis, tissue remolding, and associated renal dysfunction^2,42^. During these deterioration processes, podocytes and mesangial cells are the main targets of injury. In contrast, treatment with antioxidants effectively inhibits oxidative and inflammatory responses in these cells and thereby improves renal function^43,44^. In the present study, we found that oxidative stress and inflammation, the two major pathogenic factors in diabetes- and ADR-induced CKD, were largely abolished by FGF1^ΔHBS^ treatment. In vitro studies showed that these protective effects occurred in podocytes and mesangial cells. Taken together, these data suggest that the ameliorating effects of FGF1^ΔHBS^ on CKD are mediated by its inhibitory effects on oxidative stress and inflammation.

As a downstream target of PI3K, AKT plays a pivotal role in several cellular processes, including cell proliferation, apoptosis, glucose metabolism, cell migration, and transcription^39,45^. AKT activity is impaired in several types of CKD, leading to glomerular lesion development, podocyte injury, and CKD progression^46,47^. In podocytes, AKT dysfunction is associated with an inflammatory response and apoptosis^48^. In contrast, increased AKT signaling reduces cellular oxidative stress and inhibits the release of proinflammatory cytokines^48^. Consistent with these findings, we found that AKT phosphorylation levels were decreased in DN and AN, accompanied by increased oxidative stress and inflammation. Administration of FGF1^ΔHBS^ markedly restored the normal function of AKT and inhibited ROS production and the expression of inflammatory cytokines; all these effects were compromised by AKT siRNA or the AKT antagonist. Therefore, we conclude that FGF1^ΔHBS^ suppresses oxidative stress and inflammation and improves renal function via activation of AKT signaling.

The dysregulation of GSK-3β has been implicated in various kidney diseases^49,50^. GSK-3β is vital for the deactivation of Nrf2 and the initiation of oxidative injury; its activity is blocked via AKT activation^49,51,52^. The inhibition of GSK-3β activity has been closely associated with attenuated podocyte injury^50,53^. In the present study, we found that FGF1^ΔHBS^ activated AKT, Nrf2, and SOD2, whereas GSK-3β activity was inhibited. In contrast, AKT inhibition led to enhanced GSK-3β activity and abolition of FGF1^ΔHBS^-mediated antioxidative capacity in podocytes and mesangial cells.

ASK1 is a key factor in the initiation and development of inflammation that can also be inhibited by AKT^31,54,55^. ASK1 activates P38/JNK signaling cascades, thereby promoting inflammation and insulin resistance, whereas its deactivation significantly inhibits diabetic glomerulosclerosis and reduces renal dysfunction^56–59^. We found that both hyperglycemia and ADR enhanced the activities of ASK1 and JNK and upregulated the expression levels of profibrotic and proinflammatory signals, which could be reversed by FGF1^ΔHBS^. These data suggest that AKT is a key regulator of FGF1^ΔHBS^, mediating its ability to normalize renal function by restoring cellular redox homeostasis via the GSK-3β/Nrf2 signaling cascade and by suppressing inflammatory responses via inhibition of ASK1-mediated JNK activation.

In summary, the present study confirms the protective effect of rFGF1^ΔHBS^ in two types of CKD. Mechanistic analyses suggest that activation of PI3K/AKT signaling plays a vital role in mediating the beneficial effects of rFGF1^ΔHBS^ via upregulating the GSK-3β/Nrf2 pathway and inhibiting the ASK1/JNK signaling pathway (Fig. 8f). Our data suggest that constructing structure-based FGF1 mutants to reduce mitogenic activity is a strategy for modifying FGF1 for the treatment of CKD associated with oxidative stress and inflammation. This study also provides an applicable model for engineering other growth factors with therapeutic potential.

## Materials and methods

### Regents and antibodies

Doxorubicin (adriamycin) was purchased from Sigma (Cat# D1515), and the PI3K/AKT selective inhibitor MK-2206 was purchased from Selleck (Cat# S1078). AKT selective siRNA was purchased from Santa Cruz (Cat# 43610). The following antibodies were used to detect the proteins of interest: phospho-AKT (Cell Signaling Technology; Cat# 4060; dilution: 1:1000), AKT (Cell Signaling Technology; Cat# 4691; dilution: 1:1000), phospho-ASK (Cell Signaling Technology; Cat# 3765; dilution: 1:1000), ASK (Cell Signaling Technology; Cat #3765; dilution: 1:1000), phospho-JNK (Cell Signaling Technology; Cat# 4668; dilution: 1:1000), JNK (Cell Signaling Technology; Cat# 9258; dilution: 1:1000), COL 4 (Abcam; Cat# ab6586; dilution: 1:1000), TGF-β1 (Abcam; Cat# ab92486; dilution: 1:800), phospho-GSK-3β (Cell Signaling Technology; Cat# 2118; dilution: 1:1000), GSK-3α/β (Cell Signaling Technology; Cat# 5676; dilution: 1:1000), Nrf2 (Abcam; Cat# ab62352; dilution: 1:1000), SOD2 (Abcam; Cat# ab13533; dilution: 1:1000), NQO1 (Abcam; Cat# ab34173; dilution: 1:1000), GAPDH (Cell Signaling Technology; Cat# 2118; dilution: 1:1000), and CD68 (Abcam; Cat# ab125212; dilution: 1:1000).

### Protein expression and purification

The heparin binding mutant construct of FGF1 (FGF1^ΔHBS^) were created by sequentially introducing the Lys127Asp, Lys128Gln, and Lys133Val into the FGF1^WT^ expression construct using the QuikChange XL site-directed mutagenesis kit (Stratagene, La Jolla, CA). Expression and purification of FGF1^WT^ and FGF1^∆HBS^ were performed as previous described^22^.

### Cell culture

The conditionally immortalized mouse podocyte cell line was a gift from Dr Stuart Shankland (University of Washington). The cells were grown in plates precoated with collagen I (BD Biosciences) and RPMI-1640 medium (5.5 mM d-glucose, Gibco, Eggenstein, Germany) containing 10% FBS (Gibco, Grand Island, NY) and interferon gamma (50 U/mL, Sigma) at 33 °C. Cell differentiation was induced by incubation without interferon gamma for 10 days at 37 °C.

Mesangial cells (SV40 MES 13, ATCC-CRL-1927) were purchased from American Type Culture Collection (ATCC, Manassas, VA). The cells were maintained in Dulbecco’s modified Eagle’s medium (5.5 mM d-glucose, Gibco, Eggenstein, Germany) supplemented with 10% FBS (Gibco, Grand Island, NY) and 100 U/mL penicillin and streptomycin.

For intracellular signaling assays or DHE staining, cells were starved for 12 h in serum-free RPMI-1640 medium and pretreated with serum-free medium containing rFGF1^ΔHBS^ (10 or 100 ng/mL) or rFGF1^ΔHBS^ plus MK-2206 (3, 10, or 30 μM) for 1 h. Then, the cells were incubated in high glucose (HG, 25 mM) (with d-mannitol as an osmotic control) or ADR (0.5 μg/mL) for 12 h. Subsequently, the cells were lysed to measure the levels of various downstream signals by western blotting or were fixed for DHE staining.

For knockdown of AKT expression by siRNA, the cells were seeded in six well plates and grown to 70% confluence over 24 h. Transient transfections were performed using the transfection reagent Lipofectamine RNAiMAX (Invitrogen) according to the manufacturer’s protocol. After cells were transfected with control or AKT siRNA for 24 h, they were starved and treated as described above.

### RNA-sequencing

RNA-Seq was performed as previously described^60^. Briefly, MPCs were starved for 12 h, pretreated with rFGF1^WT^ or rFGF1^ΔHBS^ (100 ng/mL) for 1 h and then incubated with high glucose (25 mM) for another 12 h. Total RNA was extracted using TRIzol reagent (Invitrogen) following the manufacturer’s protocol. Samples of total RNA with RIN > 7.0 were processed for further analysis after evaluation using an RNA 6000 Nano LabChip Kit (Agilent). Approximately 10 µg of total RNA from MPCs was subjected to poly(A) mRNA isolation using poly-T oligo-labeled magnetic beads (Invitrogen). Following purification, mRNA was fragmented into small pieces using divalent cations at elevated temperature. Then, the RNA fragments were reverse transcribed to create a cDNA library according to the protocol provided by the manufacturer (mRNA Sequencing Sample Preparation Kit, Illumina). The average insert size for the paired-end libraries was 300 bp (±50 bp). Subsequently, we performed paired-end sequencing using an Illumina HiSeq 4000 device (LC Sciences) following the manufacturer’s protocol. Differential expression analysis was performed based on adjusted *p*-values, with volcano plots showing differences in fold change in gene expression. The threshold values for statistical significance in the volcano plots were *q* (adjusted *P* value) < 0.05 and fold change (FC) ≥2 or ≤0.5. The application edgeR was used to summarize the fragments per kilobase of exon per million reads mapped based on the equation ${\mathrm{FPKM}} = \frac{{{\mathrm{Total}}\,{\mathrm{exon}}\,{\mathrm{fragments}}}}{{{\mathrm{Mapped}}\,{\mathrm{reads}}\left( {{\mathrm{millions}}} \right) \times {\mathrm{Exon}}\,{\mathrm{length}}({\mathrm{kb}})}}$. Pathway analysis was conducted with ggplot2 as previously described^61^. Briefly, networks of these genes were generated based on their connectivity and were aligned against the KEGG (http://www.genome.jp/kegg/). The enriched genes identified by KEGG analysis were analyzed using Fisher’s exact test and the χ^2^ test, and the *p* value was corrected and adjusted to obtain the *q* value. A significant pathway was considered if the *q* value was <0.05. Raw sequencing data were submitted to GEO (GSE125693).

### Experimental protocol for the animal study

Eight-week-old male *db/db* (C57BLKS/J-*lepr*^*db*^*/lepr*^*db*^) mice, their nondiabetic *db/m* littermates and male BALB/c mice were purchased from the Model Animal Research Center of Nanjing University (Nanjing, China). All animals were acclimatized to our laboratory environment before use and were housed in a controlled environment (22 ± 2 °C, 50–60% humidity, 12-h light/dark cycle, lights on at 7 AM) with free access to food and water. The experiments were performed in accordance with the National Institutes of Health guidelines and with approval from the Animal Care and Use Committee of Wenzhou Medical University, China.

For the DN model, *db/db* mice were i.p. injected with FGF1^ΔHBS^ at 0.5 mg/kg body weight every other day for 12 weeks. *db/m* and *db/db* mice were treated with 0.9% normal saline as controls. Body weight and blood glucose levels were measured every three days. Plasma glucose levels were measured using a FreeStyle complete blood glucose monitor (Abbott Diabetes Care Inc., Alameda, CA).

For the AN model, BALB/c mice were injected with ADR (10.5 mg/kg) through the tail vein. rFGF1^ΔHBS^ (0.5 mg/kg body weight) or normal saline was given i.p. every other day starting one week prior to ADR injection and lasting for 5 weeks.

After the final injection of rFGF1^ΔHBS^ or vehicle, mouse urine was collected for 24 h in metabolic cages (TSE Systems, MO). Then, the mice were anesthetized with amobarbital sodium and sacrificed by cervical dislocation. Blood samples and kidneys were collected for subsequent analyses. BUN (C013–2, Jiancheng, Nanjing, China), ALB (E99–134, Bethyl Laboratories, Texas), and creatinine (DICT-500, BioAssay Systems, CA) levels in serum were measured using assay kits according to the manufacturer’s instructions. MDA content in renal tissue was measured according to the manufacturer’s instructions (S0131, Beyotime, Shanghai, China).

### Pathological, histopathological, immunohistochemical, and immunofluorescence evaluation of mouse kidneys

Excised kidneys were fixed in 4% paraformaldehyde for 24 h and embedded in paraffin. After deparaffinization and rehydration, paraffin sections (5 μm) were stained with H&E, Masson’s trichrome, or PAS. Assessment of glomerular mesangial expansion was performed using a semiquantitative scoring system as follows: 0, 0%; 1, <25%; 2, 25–50%; 3, 50–75%; and 4, >75%^19^.

For transmission electron microscopy, cortical kidney tissues were cut into small pieces (1 mm^3^) and fixed with 0.1 M phosphate buffer containing 2.5% glutaraldehyde and 1% tannic acid at 4 °C for 2 h. Then, these slices were treated with 1% osmium tetroxide. After dehydration, the slices were embedded in epoxy resin. Ultrathin sections were counterstained with 2% uranyl acetate and lead citrate. Conventional electron micrographs were obtained using a Philips CM10 electron microscope (Eindhoven, the Netherlands), and the average podocyte foot process width was calculated: FPW = (Π/4) × (Σ glomerular basement membrane length/Σ number of foot processes).

For immunohistochemistry, deparaffinized, and rehydrated paraffin sections (5 μm) were incubated with rabbit anti-WT-1 antibody (Abcam, ab89901, 1:400) overnight at 4 °C. After washing, the kidney sections were incubated with biotinylated goat anti-rabbit antibody (Zhongshan Golden Bridge ZB-2010, 1:100) for 1 h. After four washes with PBS, the sections were stained with 4′,6-diamidino-2-phenylindole (DAPI) (Beyotime, China) for 10 min and visualized using a microscope (Nikon, Tokyo, Japan).

For immunofluorescence, frozen sections (5 μm) of renal tissue were incubated with rabbit anti-FGF1 antibody (Abcam; Cat# ab207321, 1:200) or rat anti-F4/80 (Abcam; Cat# ab6640, 1:200) overnight at 4 °C, followed by incubation with secondary antibody (goat anti-rabbit-IgG Alexa Fluor 488, Invitrogen, A11001, USA, 1:1000) for 1 h (not for F4/80 staining). Thereafter, the sections were incubated with DAPI (SouthernBiotech, Birmingham, AL) for 10 min, and immunofluorescence was analyzed using a confocal microscope (Leica, Mannheim, Germany).

For DHE staining, frozen sections (5 μm) of renal tissues or cultured cells were incubated with DHE (Beyotime; Cat# S0063, 1.5 mmol/L) for 30 min and visualized using a fluorescence microscope (TCS-SP8, Leica, Germany).

### Western blot analysis

Renal tissues (30–50 mg) or cells were lysed with RIPA lysis buffer (25 mM Tris, pH 7.6, 150 mM NaCl, 1% NP-40, 1% sodium deoxycholate, and 0.1% SDS) containing protease and phosphatase inhibitors (Thermo Fisher Scientific, US), and protein concentration was determined using the Bradford protein assay kit (Bio-Rad, CA). After normalization, equal amounts of protein were separated by 12% SDS–PAGE and transferred to PVDF membranes (0.45 μm, Millipore, Germany). The membranes were blocked with 10% nonfat milk in TBST for 2 h and incubated with primary antibodies at 4 °C overnight. After three washes with TBST, the membranes were incubated with HRP‐conjugated secondary antibodies (Cell Signaling; #7074 or #7076, 1:3000) at room temperature for 1 h. Then, the blots were incubated using the EasySee western Blot Kit (Transgen Biotech, China) to visualize the immunoreactive bands. Densitometric analysis was performed using ImageJ software version 1.38e (NIH, USA).

### RNA extraction, cDNA synthesis, and quantitative RT-PCR

Total RNA extraction and quantitative reverse transcription polymerase chain reaction were performed as previously described^19^. Briefly, total RNA was extracted from renal tissues or cultured cells using TRIzol reagent (Invitrogen, Shanghai, China) and reverse transcribed using the HiScript II 1st Strand cDNA Synthesis Kit (Vazyme R212–01, Nanjing, China). Real-time PCR was performed using the QuantStudio3 system with AceQ Universal SYBR qPCR Master Mix (Vazyme Q511–02, Nanjing, China). All data were normalized to the expression of the Ubc gene. The sequences of the PCR primers are provided in Table S1.

### Statistical analysis

The in vitro experiments were performed three times with triplicate samples for each individual experiment. Data obtained from the animal study were obtained from five to eight mice. All data are expressed as the mean ± SEM. Comparisons among groups were performed using one-way ANOVA, followed by Duncan’s multiple range test for differences between two groups; Student’s *t* test was used when appropriate. A value of *p* < 0.05 was considered significant.

## Supplementary information

Supplemental information

Table supplemental 1
