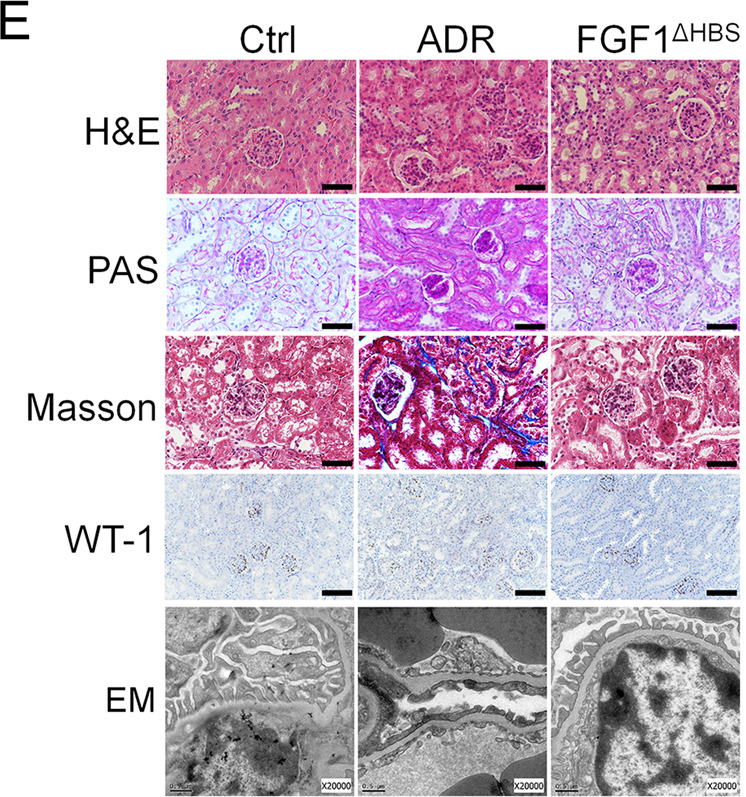# Correction: FGF1^ΔHBS^ ameliorates chronic kidney disease via PI3K/AKT mediated suppression of oxidative stress and inflammation

**DOI:** 10.1038/s41419-023-05808-x

**Published:** 2023-04-19

**Authors:** Dezhong Wang, Mengyun Jin, Xinyu Zhao, Tianyang Zhao, Wei Lin, Zhengle He, Miaojuan Fan, Wei Jin, Jie Zhou, Lingwei Jin, Chao Zheng, Hui Jin, Yushuo Zhao, Xiaokun Li, Lei Ying, Yang Wang, Guanghui Zhu, Zhifeng Huang

**Affiliations:** 1 School of Pharmaceutical Sciences & Center for Structural Biology, Wenzhou Medical University, Wenzhou, 325035 Zhejiang China; 2 School of Life and Environmental Science, Wenzhou University, Wenzhou, 325035 Zhejiang China; 3 The Second Affiliated Hospital and Yuying Children’s Hospital of Wenzhou Medical University, Wenzhou, 325035 Zhejiang China; 4 School of Basic Medical Sciences, Wenzhou Medical University, Wenzhou, 325035 Zhejiang China; 5 The First Affiliated Hospital of Wenzhou Medical University, Wenzhou, 325035 Zhejiang China

Correction to: *Cell Death and Disease* 10.1038/s41419-019-1696-9, published online 12 June 2019

The original version of this article contained an error in Figure 5E. The representative image of transmission electron microscope (EM) analysis for FGF1ΔHBS treatment group was accidentally assigned to the Control group when the authors prepared this figure in PowerPoint (bottom line of Figure 5E). The relevant results previously reported as statistically significant remain to be unchanged. The authors note that this mistake doesn’t affect the conclusion in this study. The correct image is given below.