# Supplementary material for: FGF1ΔHBS ameliorates chronic kidney disease via PI3K/AKT mediated suppression of oxidative stress and inflammation
Source: Cell Death Dis. 2019 Jun 12;10(6):464. doi: 10.1038/s41419-019-1696-9 (PMC6561918; doi:10.1038/s41419-019-1696-9)
Supplement: Supplementary file 1 — Supplemental information [file 41419_2019_1696_MOESM1_ESM.docx]

**SUPPLYMENTARY INFORMATION**

# FGF1^△HBS^ Ameliorates Chronic Kidney Disease via PI3K/AKT Mediated Suppression of Oxidative Stress and Inflammation


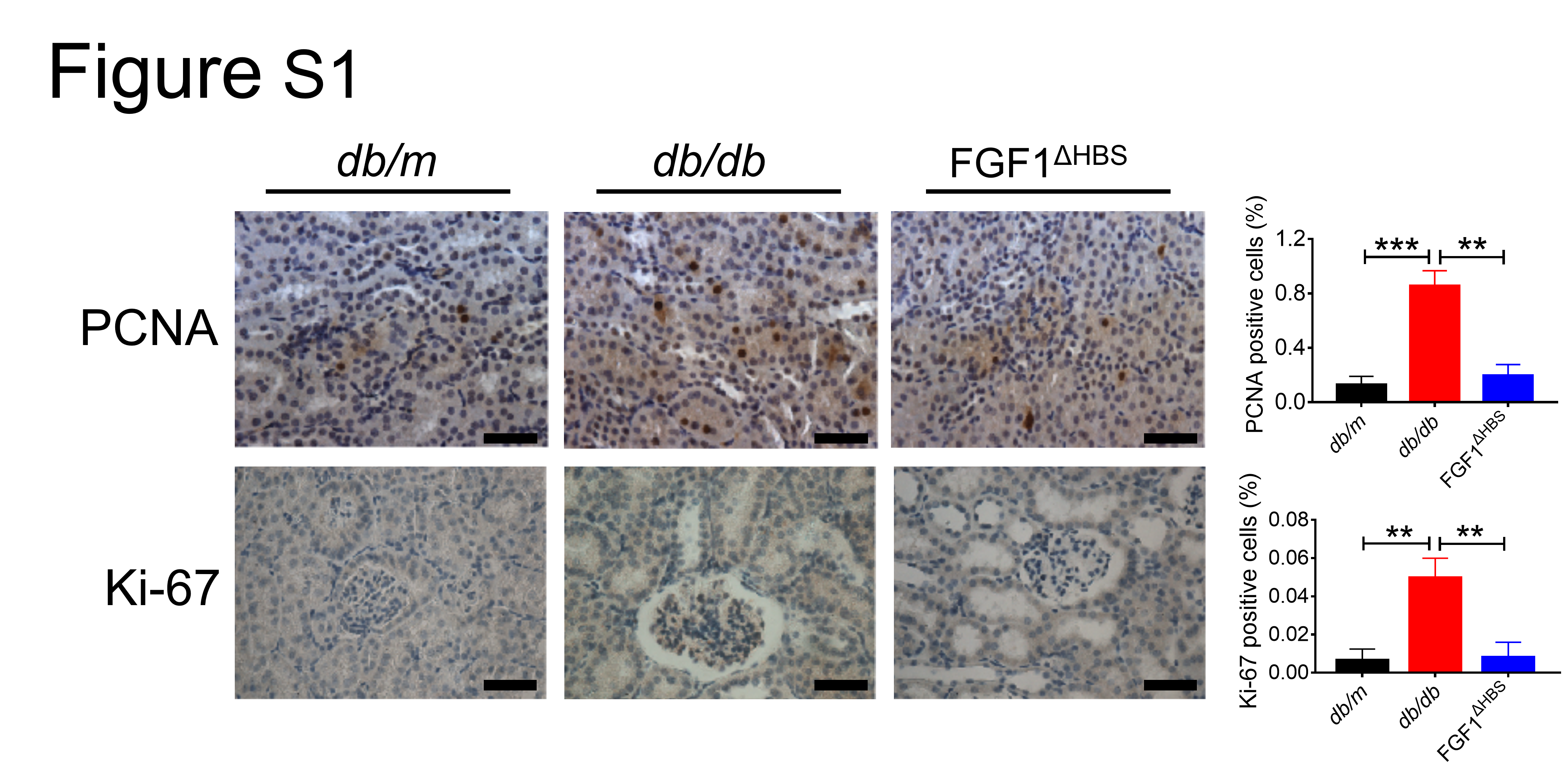


**Figure S1. Mitogenic activity of rFGF^ΔHBS^ on renal tissues of *db/db* mice.** Immunohistochemical staining with antisera to PCNA or Ki-67 in the renal tissue of *db/db* mice after 12 weeks of chronic administration of rFGF1^ΔHBS^ (0.5 mg/kg body weight) or control vehicle. Data are presented as the mean ± SEM (n = 8); ***p*<0.01, ****p*<0.001*.* Scale bars, 50 μm.

**
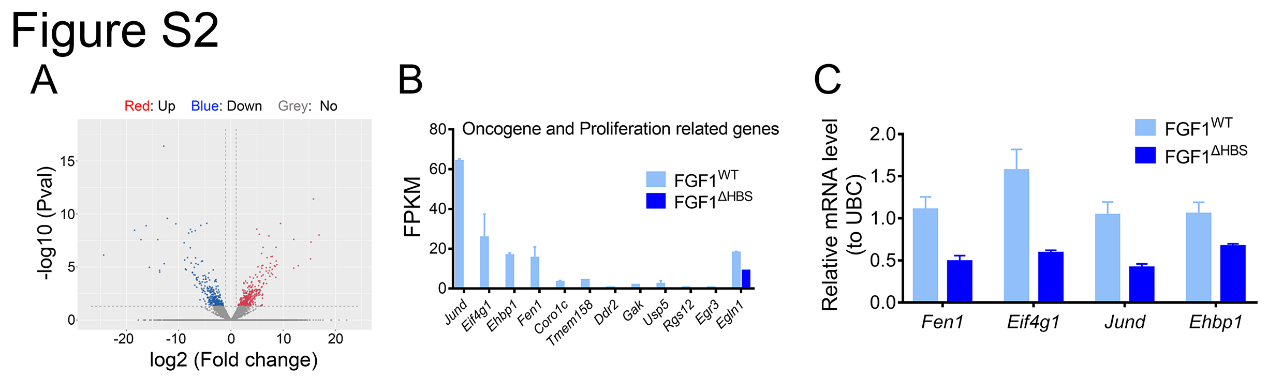
**

**Figure S2. rFGF1^ΔHBS^ displays reduced ability to activate proliferative genes.**  **(A)** Global distribution of expressed genes as revealed by volcano plots with adjusted Pval to label Y axis. **(B)** FPKM of selected genes related to proliferation (q<0.05 and fold change (FC) ≥ 2 or ≤ 0.5)**. (C)** Real-time PCR analysis of *Fen1*, *Eif4g1*, *Jund* and *Ehbp1* mRNAs in mouse podocyte treated with HG (25 mM), HG plus rFGF1^WT^ (100 ng/mL) or HG plus rFGF1^ΔHBS^ (100 ng/mL).


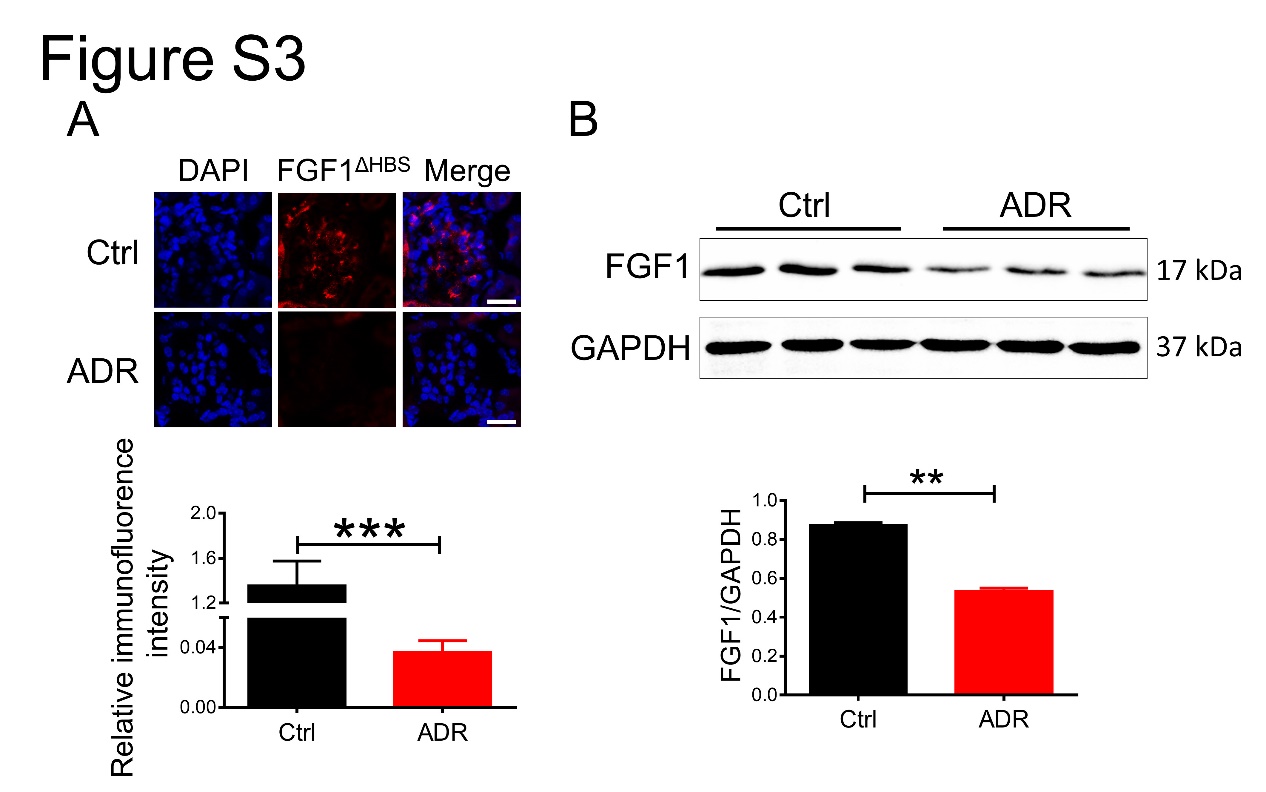
**Figure S3. FGF1 is decreased in renal tissues of ADR- induced nephropathy mice. (A, B)** Analysis of renal tissue from ADR-induced (10.5 mg/Kg body weight) nephropathy mice. **(A)** Immunofluorescence staining of FGF1(red); Scale bars, 50 μm. **(B)** Protein expression of FGF1 as determined by Western blot analysis (upper panel) and quantitation using ImageJ software (lower panel). Data are presented as the mean ± SEM (n = 8). ***p*<0.01, ****p*<0.001*.*

**
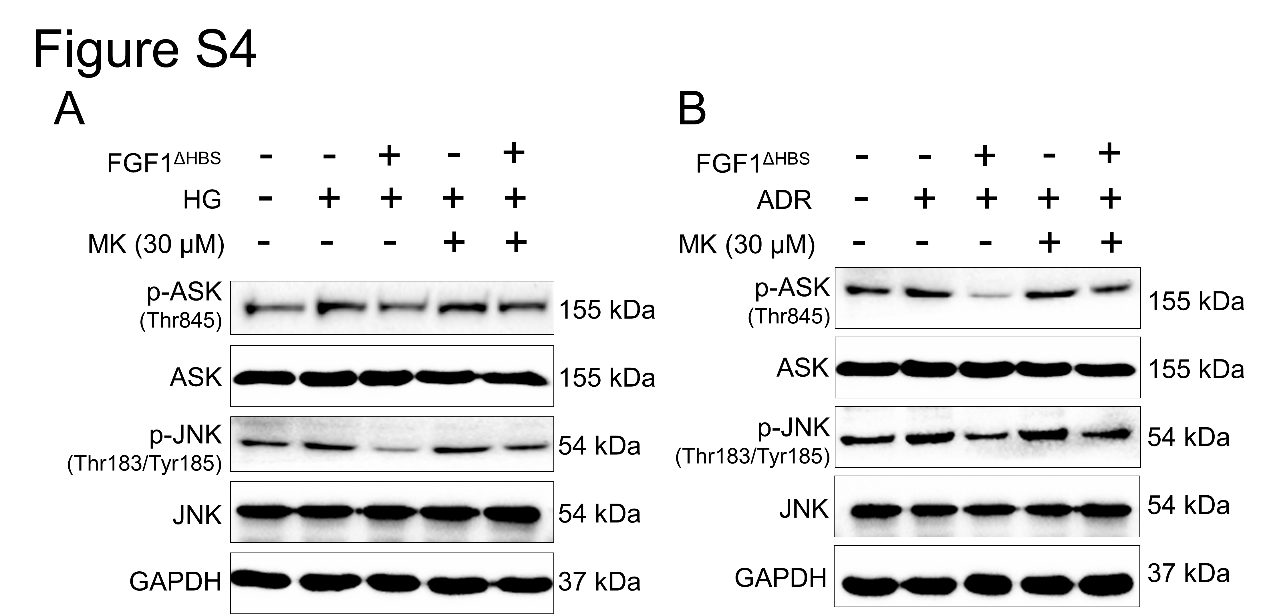
Figure S4. AKT suppresses HG-induced ASK1 and JNK activations. (A, B)** Phosphorylation levels of ASK and JNK in mouse podocytes pretreated with rFGF1^ΔHBS^ (100 ng/mL) or rFGF1^△HBS^ plus MK-2206 (30 μM) for 1 hr and exposed to high glucose (HG, 25mM) (A) or adriamycin (0.5 μg/mL) (B) for additional 12 hrs as determined by Western blot analysis.


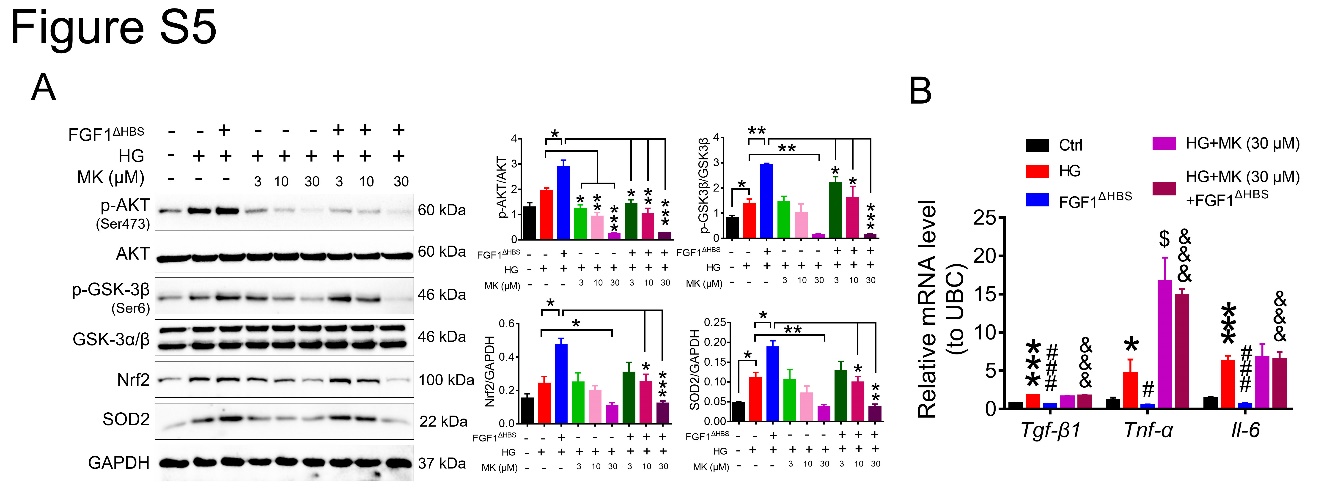


**Figure S5. AKT mediates the anti-oxidative and anti-inflammatory capacity of FGF1^ΔHBS^ on mouse podocyte cells under high glucose challenge.** Analysis of mouse podocytes pretreated with rFGF1^ΔHBS^ (100 ng/mL) or rFGF1^△HBS^ plus MK-2206 (3, 10, or 30 μM) for 1 h and exposed to either low glucose (LG) or high glucose (HG) (25 mM) for additional 12 hrs. **(A)** Phosphorylation levels of AKT and GSK-3β and protein expressions of Nrf2, NQO1 and SOD2 as determined by Western blot analysis (left panel) and quantitation using ImageJ software (right panel). **(B)** Real-time PCR analysis of expression of *TGF-β1, Tnf-α,* and *IL-6* mRNAs. Data from three independent measurements are presented as the mean ± SEM. Panel A, **p* < 0.05, ***p* < 0.01, ****p* < 0.001; panel B, **p* < 0.05, ****p* < 0.001 vs Ctrl; ^#^*p* < 0.05, ^###^*p* < 0.001 vs HG; ^$^*p* < 0.05 vs HG; ^&&&^*p* < 0.001 vs HG+FGF1^ΔHBS^.


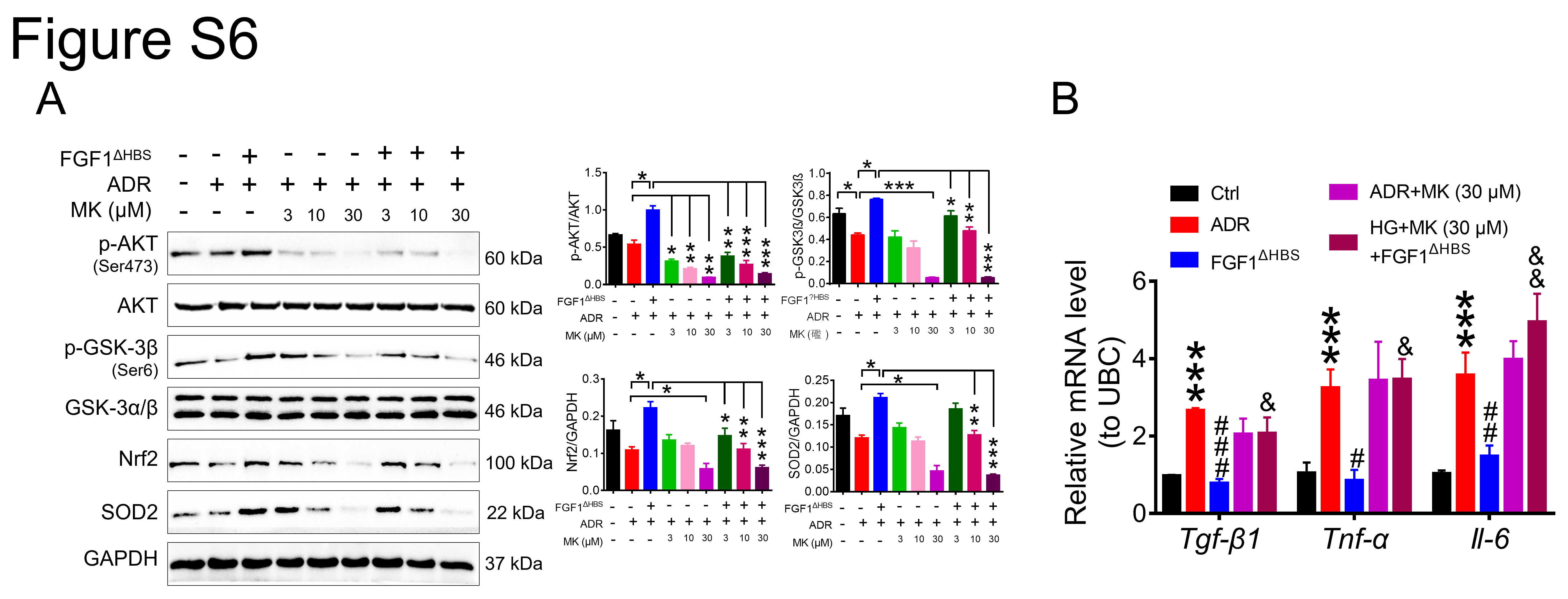


**Figure S6.** **AKT mediates the anti-oxidative and anti-inflammatory capacity of FGF1^ΔHBS^ on mouse podocyte cells under adriamycin** **challenge.** Analysis of mouse podocytes pretreated with rFGF1^ΔHBS^ (100 ng/mL) alone or rFGF1^△HBS^ (100 ng/mL) plus MK-2206 (3, 10, or 30 μM) for 1 hr and exposed to ADR (0.5 μg/mL for 12 hrs). **(A)** Phosphorylation levels of AKT and GSK-3β and protein expressions of Nrf2, NQO1 and SOD2 as determined by Western blot analysis (left panel) and quantitation using ImageJ software (right panel); **(B)** Real-time PCR analysis of expression of *TGF-β1, Tnf-α,* and *IL-6* mRNAs. Data from three independent measurements are presented as the mean ± SEM. Panel D, **p* < 0.05, ***p* < 0.01, ****p* < 0.001; panel E, ^***^*p* < 0.001 vs Control; ^#^*p* < 0.05, ^##^*p* < 0.01, ^###^*p* < 0.001 vs HG; ^&^*p* < 0.05; ^&&^*p* < 0.001 versus HG+FGF1^ΔHBS^*.*

**
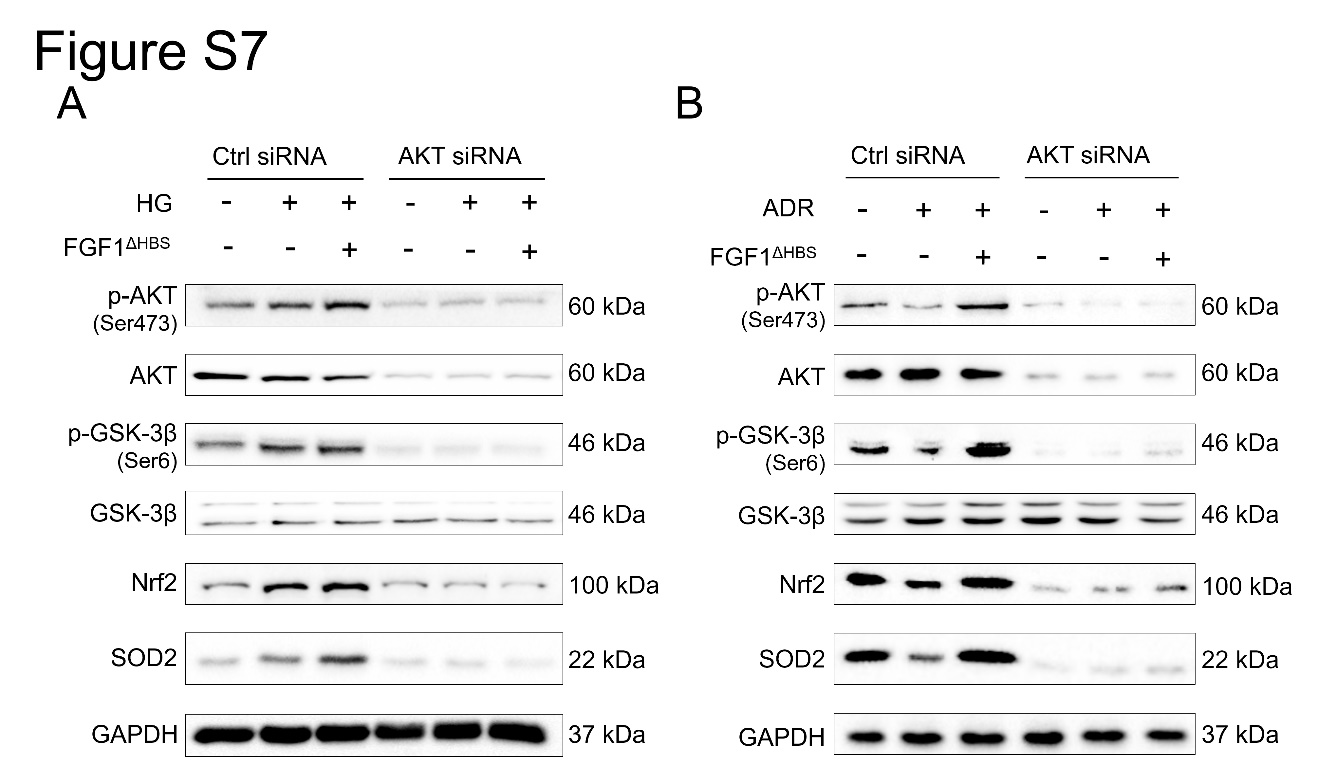
**

**Figure S7. AKT mediates the anti-oxidative capacity of FGF1^ΔHBS^ on mouse mesangial cells.** Cells were transfected with control or AKT siRNA, pretreated with rFGF1^ΔHBS^ (100 ng/mL) for 1 hr and incubated with high glucose (HG, 25 mM) (A) or adriamycin (0.5 μg/mL) (B) for additional 12 hrs. **(A, B)** Phosphorylation levels of AKT and GSK-3β and protein expression of Nrf2 and SOD2 as determined by Western blot analysis.
