# Supplementary material for: FGF1ΔHBS ameliorates chronic kidney disease via PI3K/AKT mediated suppression of oxidative stress and inflammation
Source: Cell Death Dis. 2019 Jun 12;10(6):464. doi: 10.1038/s41419-019-1696-9 (PMC6561918; doi:10.1038/s41419-019-1696-9)
Supplement: Supplementary file 2 — Table supplemental 1 [file 41419_2019_1696_MOESM2_ESM.docx]

**Table S1.** Primers used in this study

| Primer Name | Sequence (5’->3’) |
| --- | --- |
| Sod2-F | CAGACCTGCCTTACGACTATGG |
| Sod2-R | CTCGGTGGCGTTGAGATTGTT |
| Nqo1-F | ATGGGAGGTGGTCGAATCTGA |
| Nqo1-R | GCCTTCCTTATACGCCAGAGATG |
| Nfe2l2-F | TCTTGGAGTAAGTCGAGAAGTGT |
| Nfe2l2-R | GTTGAAACTGAGCGAAAAAGGC |
| Cat-F | TGGCACACTTTGACAGAGAGC |
| Cat-R | CCTTTGCCTTGGAGTATCTGG |
| Catalase-F | TGGCACACTTTGACAGAGAGC |
| Catalase-R | CCTTTGCCTTGGAGTATCTGG |
| Ehbp1-F | CAGCGTGTGGGAAAACATGC |
| Ehbp1-R | TGTCCAAACAACCACCAGTTTA |
| Fen1-F | CATCAAGCCTGTGTACGTCTT |
| Fen1-R | TGGGTGCATCAAGGTAAGGGA |
| Eif4g1-R | TGTTCTCGGTGCTCTTCCATC |
| Eif4g1-F | AAGACCTCATCTCGCATCCG |
| Jund-F | CCATCGACATGGACACGCAA |
| Jund-R | CAGCTCGGTGTTCTGGCTTT |
| G6pc2-F | CAGGAGGACTACCGGACTTAC |
| G6pc2-R | TCAACTGAAACCAAAGTGGGAA |
| Foxo3-F | CTGGGGGAACCTGTCCTATG |
| Foxo3-R | TCATTCTGAACGCGCATGAAG |
| Bcl2l1-F | AGGCGATGAGTTTGAACTGC |
| Bcl2l1-R | TGAAGCTGGGATGTTAGATCACT |
| Lama1-F | CAGCGCCAATGCTACCTGT |
| Lama1-R | GGATTCGTACTGTTACCGTCACA |
| Il-33-F | TCCAACTCCAAGATTTCCCCG |
| Il-33-R | CATGCAGTAGACATGGCAGAA |
| Cxcl12 -F | TGCATCAGTGACGGTAAACCA |
| Cxcl12 -R | TTCTTCAGCCGTGCAACAATC |
| Lbp-F | GATCACCGACAAGGGCCTG |
| Lbp-R | GGCTATGAAACTCGTACTGCC |
| Il-6-F | TAGTCCTTCCTACCCCAATTTCC |
| Il-6-R | TTGGTCCTTAGCCACTCCTTC |
| Il-1β-F | GCAACTGTTCCTGAACTCAACT |
| Il-1β-R | ATCTTTTGGGGTCCGTCAACT |
| IL17rb-F | AGCCGACTATTCAGTGTGGC |
| IL17rb-R | GTCTTGACGAGTTCCACTTGG |
| Fn1-F | GCAGTGACCACCATTCCTG |
| Fn1-R | GGTAGCCAGTGAGCTGAACAC |
| Acta2-F | CCCAACTGGGACCACATGG |
| Acta2-R | TACATGCGGGGGACATTGAAG |
| Tnf-α-F | GACGTGGAACTGGCAGAAGAG |
| Tnf-α-R | TTGGTGGTTTGTGAGTGTGAG |
| Tgf-β1-F | CTCCCGTGGCTTCTAGTGC |
| Tgf-β1-R | GCCTTAGTTTGGACAGGATCTG |
| Ubc-F | GCCCAGTGTTACCACCAAGA |
| Ubc-R | CCCATCACACCCAAGAACA |
